# Thick Sintered Electrode Lithium-Ion Battery Discharge Simulations: Incorporating Lithiation-Dependent Electronic Conductivity and Lithiation Gradient Due to Charge Cycle

**DOI:** 10.1149/1945-7111/abc747

**Published:** 2020-11-16

**Authors:** Chen Cai, Ziyang Nie, J. Pierce Robinson, Daniel S. Hussey, Jacob M. LaManna, David L. Jacobson, Gary M. Koenig

**Affiliations:** 1 Department of Chemical Engineering, University of Virginia, Charlottesville 22904-4741, Virginia, United States of America; 2 National Institute of Standards and Technology Physical Measurements Laboratory, Gaithersburg, 20899-8461 Maryland, United States of America

## Abstract

In efforts to increase the energy density of lithium-ion batteries, researchers have attempted to both increase the thickness of battery electrodes and increase the relative fractions of active material. One system that has both of these attributes are sintered thick electrodes comprised of only active material. Such electrodes have high areal capacities, however, detailed understanding is needed of their transport properties, both electronic and ionic, to better quantify their limitations to cycling at higher current densities. In this report, efforts to improve models of the electrochemical cycling of sintered electrodes are described, in particular incorporation of matrix electronic conductivity which is dependent on the extent of lithiation of the active material and accounting for initial gradients in lithiation of active material in the electrode that develop as a consequence of transport limitations during charging cycles. Adding in these additional considerations to a model of sintered electrode discharge resulted in improved matching of experimental cell measurements.

Increased development and proliferation of portable electronic devices and electric vehicles has led to demands for increases in the performance and in particular the energy density for the batteries that power them. Lithium (Li)-ion batteries have been the prevailing option in these applications due to the intrinsically high volumetric and gravimetric energy density for the relevant materials chemistry available for these technologies.^1^ However, in addition to materials chemistry, electrode design and composition can have a significant effect on cell energy density, particularly on a volumetric basis. For example, increased electrode thickness will generally result in higher energy density batteries at the cell level due to the reduced relative fraction of inactive components, although at increased thicknesses increased resistance due to transport limitations results in reduced power density and rate capability.^2^

Recently, there have been reports of Li-ion electrodes where the electrodes contain only active material sintered together.^3–7^ Such electrodes can be made very thick (even >1 mm^6^), enabling very high areal capacities which translates to high energy density at the cell level. The lack of binder and high surface area conductive additives can improve Li^+^ transport properties through the electrode microstructure, however, at such large thicknesses mass transport limitations will still limit the rate capability and current densities batteries containing thick sintered electrodes can achieve.^5,7^ Recent *in operando* neutron imaging experiments with thick sintered electrodes provided evidence further supporting Li^+^ transport in the electrolyte phase through the electrode microstructure limiting the rate capability of batteries containing these electrodes,^7^ and experimental results were compared to simulations using the 1-D model based on Newman et al.^8^

The model previously employed largely captured the qualitative polarization and Li^+^ redistribution characteristics during discharge that were observed experimentally,^5,7^ however, there were significant quantitative differences in the polarization curves. These differences were in part speculated to be due to the assumption of a single electronic conductivity for the electrode matrix in the sintered electrodes. For conventional composite electrodes, a single constant matrix electronic conductivity is a reasonable assumption because most of the electron conduction through the matrix would be expected to proceed through the conductive additive-laden (e.g., carbon black) composite phase within the interstitial region between active material particles.^8,9^ However, for sintered electrodes there were no conductive additives, and thus the electronic conduction must proceed through the active material itself, including interparticle connections. For the system considered in this manuscript and previously reported,^7^ the anode material was Li_4_Ti_5_O_12_ (LTO) and the cathode material was LiCoO_2_ (LCO). For both LTO and LCO, the electronic conductivity is dependent on the extent of lithiation of the active material.^10–12^ Thus, incorporating an electrode matrix conductivity that reflected the different extents of lithiation in the different regions of the electrode would be expected to result in a more accurate model and prediction of the electrochemical properties of sintered electrodes. In addition, both experimental measurements and simulations were consistent with a delithiation front that propagated in the thicker LTO anode during discharge, even at relatively low rates of discharge.^5,7^ The previous model assumed a uniform Li^+^ concentration within each electrode before the initiation of discharge. However, a Li^+^ concentration front would be expected to also occur during charging of the cell, which would result in a gradient in the Li^+^ distribution before discharge was initiated.

Herein, we report an improved 1-D model of the electrochemical discharge of sintered electrode Li-ion batteries by addressing the two previous limitations described above. A matrix conductivity will be calculated during each time point and reflecting the changes in electronic conductivity due to changes in the extent of lithiation at each point throughout the thickness of each electrode. In addition, the charging of the cell will be simulated, and the starting point of the discharge will be after the non-uniform Li^+^ distribution that resulted from the charging process. These modifications for the model resulted in improvements in the match between experiments and simulations. Improved models of sintered electrode Li-ion batteries will be needed to facilitate more rapid assessment of their performance from changes to material and cell properties to accelerate their further development.^8^

## Experimental and Simulation Methods

The model utilized an implicit numerical method to solve the system of partial differential equations (PDE) for the 1-D model. The system of equations has been described previously in the literature,^8,9,13^ however, they are also provided in the Supporting Information. For the simulations, the cathode thickness was discretized into 50 points (6.91 *μ*m between points), the anode thickness was discretized into 50 points (9.36 *μ*m between points), and the separator thickness had 10 points (2.5 *μ*m between points). At the individual particle level, the particle radius was discretized into 10 points for cathode solid particles (17 nm between points), and for the anode the radius was 10 discrete points (20 nm between points). The PDEs are a volume averaged 1-D system, as proposed by Newman.^8,9^ Although the electrodes are referred to as “sintered,” the thermal treatment was relatively mild to help retain sufficient porosity to facilitate Li^+^ transport. The initially processed particles have length scales of a few hundred nanometers and do not substantially grow during the thermal treatment, and the electrode itself does not increase in density, thus the electrodes were treated in the model as packed spherical particles with a porosity corresponding to the measured values (∼40%). Scanning electron micrographs of the electrode surfaces before cell fabrication can be found in Supporting Information, Fig. S1 (available online at stacks.iop.org/JES/167/140542/mmedia). All related model parameters can be found in Tables I and II.

To the best of our knowledge, this manuscript is the first model of a Li-ion electrode to employ a variable matrix electronic conductivity, which as mentioned earlier was a consequence of the use of sintered electrodes only containing active material. For simulations where the electronic conductivity was variable, the electronic conductivity at each discretized point within each electrode was a function of the extent of lithiation within that electrode at any given time during the discharge simulation. The LTO and LCO electronic conductivity as a function of Li^+^ content can be found in Supporting Information, Fig. S2. These values were based on literature reports.^10–12^

As described earlier, the charging profile also was simulated in this manuscript. For the charging simulation, the values of lithiation of the anode and cathode before initiating charge were x = 0.964 (for Li_x_CoO_2_) and y = 0.059 (for Li_4+3y_Ti_5_O_12_), where x and y had the same initial value at every point throughout each electrode. These initial x and y values were determined by the irreversible first cycle capacity loss of the experimental cell. While later cycles had high coulombic efficiency, the experimental first charge was 135 mAh g^−1^ LCO and the first discharge was 125 mAh g^−1^ LCO. Using the theoretical maximum for extraction of all Li^+^ from the LCO (274 mAh g^−1^ ), the initial state for the LCO was Li_0.964_CoO_2_. Balancing the capacity appropriately for the LTO electrode gave the value for y. The end point for the charge in the simulation was chosen as the charging time which resulted in the lowest error for the subsequent C/20 discharge simulation relative to the experimental polarization curve. The condition that resulted in the best match for C/20 discharge was t = 801 min of charge, which corresponded to “average” values of x ∼ 0.59 (for Li_x_CoO_2_) and y ∼ 0.66 (for Li_4+3y_Ti_5_O_12_) before initiation of discharge (the precise value for x and y varied slightly depending on the simulation case). Note that C/ 20 corresponded to a current density of 11 A m^−2^. The experimental discharge voltage profile was from a previous report.^7^

In this manuscript, three different situations for discharge simulations will be described. For sintered electrode batteries, electrodes undergo extended charge/discharge even though they have no conductive additives or composite framework.^5–7,26^ Thus, it has been assumed that electronic conductivity proceeds through the active material and its particle-particle contacts. This conduction was accounted for in the model by using the electronic conductivity of the active material (either as a constant or a function of extent of lithiation) as the matrix conductivity typically employed in previous composite porous electrode modelling.^8,20^ The first situation will use a single constant value of the matrix electronic conductivity, similar to how composite electrodes and previous studies of sintered electrodes have been treated.^5,7,20^ For this situation, the LTO and LCO were both assumed to have a fixed matrix electronic conductivity in the electrodes of 0.5 S m^−120^ This case will be referred to as “Fixed Conductivity.” In the second situation, the electronic conductivity at each point in the electrode will have a matrix electronic conductivity that varies with time/discharge and is a function of the extent of lithiation of the active material at that location. The dependence of the electronic conductivity as a function of extent of lithiation was based on literature reports, and a plot of this behavior for both LTO and LCO can be found in Fig. S2.^10–12^ This case will be referred to as “Variable Conductivity.” The Variable Conductivity case assumed an initial extent of lithiation profile that was constant within each individual electrode. For the final case, the nonuniform initial Li^+^ concentration profile in the solid phase was taken into account before the discharge simulation. This Li^+^ concentration profile was determined from first simulating the charging process for the cell. This case will be referred to as “Initial Gradient” to reflect that there was an initial nonuniform Li^+^ concentration gradient within the electrodes which resulted from the charging process of initially uniform electrode extents of lithiation. For the Initial Gradient case, the matrix electronic conductivity was dependent on extent of lithiation during both the charging and discharging simulations, using the same function as the Variable Conductivity case.

The experimental data used for comparison to the results from the model in this report were previously published.^7^ The details of material and cell construction can be found in that report. The two experimental outcomes that will be compared to the simulations in the present study are discharge polarization curves and neutron imaging analysis. For the polarization curves, the voltage profiles were captured using a potentiostat (Bio-logicSP-50^aa^Certain trade names and company products are mentioned in the text or identified in an illustration in order to adequately specify the experimental procedure and equipment used. In no case does such identification imply recommendation or endorsement by the National Institute of Standards and Technology, nor does it imply that the products are necessarily the best available for the purpose.) and the discharge currents and current densities used in the simulations were identical to those in the experiments. All other attributes of the simulation were chosen to mirror the experimental system. Neutron imaging was conducted at the thermal Neutron Imaging Facility (NIF) beamline BT-2 at the National Institute for Standards and Technology (NIST) Center for Neutron Research on the cells *in operando* while the discharge process was proceeding, and the polarization measurements were simultaneously collected. The most significant contributor to changes in neutron transmission through the battery cell are changes in the Li^+^ concentration in the solid phase in the electrodes, and thus neutron transmission changes indicate changes in the extent of lithiation within the active material at the different thickness locations within each electrode.^5,7,27^ Li^+^ is highly attenuating to neutrons, and thus increases in neutron transmission through the cell at a given location correlate to a decrease in the Li^+^ at that same location (and decreases in neutron transmission correlate to an increase in Li^+^ at those locations). Note that in the simulations both the solid phase and electrolyte phase Li^+^ concentration is calculated at different electrode depths throughout the charge/discharge process, and the total change in Li^+^ (e.g. combined solid and liquid phase weighted by their volume fractions) was compared to the changes in neutron transmission. In many situations, the changes in the solid phase Li^+^ concentration were calculated to be the most significant (>10× concentration change relative to liquid phase). More detailed discussion of Li^+^ concentration changes and neutron transmission will be described below, however, the relative change in Li^+^ concentration in the solid phase relative to the liquid phase at different cell depths for one of the cases considered in this manuscript can be found in the Supporting Information, Fig. S3. large fractions of the electrode were calculated to have >10× Li^+^ concentration change in the solid phase relative to the liquid phase, and regions that did not have very high relative contributions in the solid phase were those that experienced very low total changes in Li^+^ and thus would be expected to have relatively small changes in relative neutron transmission. Detailed descriptions of collection and processing of neutron imaging data of *in operando* coin cells can be found in previous reports.^5,7^

## Results and Discussion

### Discharge curves.—

#### Fixed Conductivity case.—

**Figure 1.**
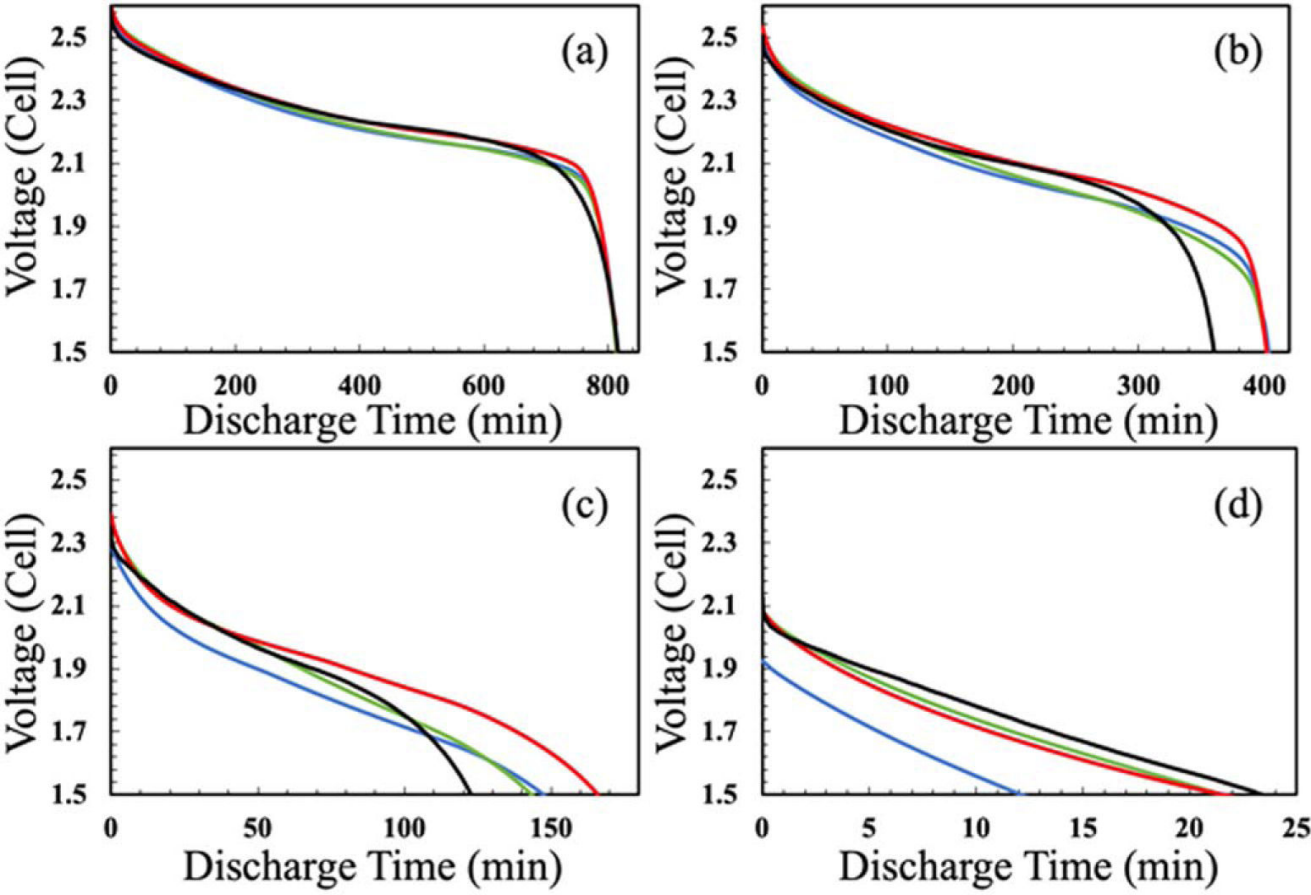
Discharge voltage profiles at discharge rates of (a) C/20 (1.1 mA cm^−2^), (b) C/10 (2.2 mA cm^−2^), (c) C/5 (4.4 mA cm^−2^), and (d) C/2.5 (8.8 mA^−2^ files on each plot correspond to the Experimental data^7^ (black) and simulation cases of Fixed Conductivity (blue), Variable Conductivity (green), and Initial Gradient (red).

For this case, both LTO and LCO were assumed to have a fixed and uniform matrix conductivity of 0.5 S m^−1^,^20^ which has been previously applied in electrode simulations using similar methods. As can be seen in Fig. 1, for all discharge rates investigated, the Fixed Conductivity case had the greatest polarization of all of the simulations and also had the greatest offset relative to the experimental discharge profile. The difference in potential relative to the experimental curve was greater at increasing discharge rates.

#### Variable conductivity case.—

As described earlier, the electronic conductivity of both LTO and LCO have previously been reported to be a function of the Li^+^ concentration within the solid phase,^10–12^ and thus conductivity as a function of extent of lithiation of the solid was incorporated into the model. At C/20 (Fig. 1a) and C/10 (Fig. 1b), the discharge voltage at early times (<50% of discharge) matches the Experimental discharge curve very closely, but at later discharge times the simulated voltage was lower than Experimental. Interestingly, the Variable Conductivity voltage was lower than the Fixed Conductivity voltage at C/20 discharge rate after ∼540 min, at C/10 discharge rate after ∼270 min, and at C/5 discharge rate after ∼130 min. This outcome will be discussed in further detail later and was a result of the Li^+^ concentration profile in the electrolyte phase. At C/5 discharge rate (Fig. 1c), the discharge curve overall matched well with the Experimental for the first ∼100 min of discharge, however, the Variable Conductivity simulation had greater final capacity/discharge time. At C/2.5 discharge rate (Fig. 1d), both the Experimental and Variable Conductivity discharge curves are nearly linear and have an excellent match early in the discharge, although the Variable Conductivity simulation has a slightly greater slope than the Experimental curve.

#### Initial gradient case.—

When looking at the first half of the Experimental discharge, the Initial Gradient simulation had the closest match of the three cases at C/20, C/10, and C/5 (Figs. 1a–1c). However, especially towards the end of the simulation the potential offset and final discharge capacity had significant deviations relative to the Experimental discharge at C/10 and C/5 (Figs. 1b, 1c). The total discharge capacity and slope of the discharge curve was a good match for the Initial Gradient simulation relative to Experimental at C/2.5 (Fig. 1d), although there was an offset in the polarization in the discharge which caused an offset between the two curves after the first few minutes.

### Changes in total Li^+^ concentration.—

**Figure 2.**
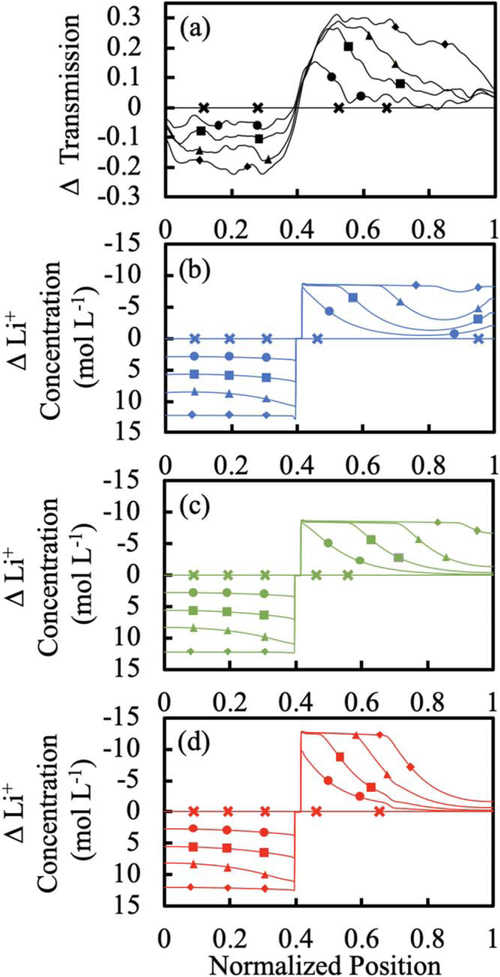
(a) Experimental changes in neutron transmission relative to the initiation of discharge at C/20 at different relative positions in the cells, where 0 corresponded to the LCO current collector and 1 corresponded to the LTO current collector.^7^ The simulated change in total Li^+^ concentration relative to the beginning of discharge at the same corresponding relative locations in the cell for discharge at the same rate using the cases of (b) Fixed Conductivity, (c) Variable Conductivity, and (d) Initial Gradient. The symbols added to the lines designate the extent of the discharge, where for each experiment/simulation the relative transmission/concentration change is shown for 0% (х), 25% (●), 50% (■), 75% (▲), and 100% (◆) of the delivered discharge capacity. Note that the symbol location has been arbitrarily chosen just for the purpose of designating the corresponding curves.

While collecting discharge curves of the Experimental cells at different rates, simultaneously neutron transmission data was also collected.^7^ The change in relative neutron transmission compared to the initiation of discharge was extracted from neutron radiographs (Fig. 2a), and these changes were predominantly due to redistribution of Li^+^ in the cell during discharge.^7^ For all discharge simulations, the Li^+^ concentration in both the solid and electrolyte phase was also extracted at each position in the cell as a function of time (Figs. 2b–2d). For comparison between the experimental data and the simulations, the total change in Li^+^ concentration was determined relative to the beginning of discharge, where the total Li^+^ concentration at each location in the cell was calculated as the electrolyte Li^+^ concentration multiplied by the porosity added to the solid phase Li^+^ concentration multiplied by (1-porosity). Note that the y-axes for total concentration profiles have been flipped (values decrease in the up direction) for more straightforward comparison with the relative changes in neutron transmission (e.g., increased transmission correlates with decreased total Li^+^ concentration). The discharge discussed in detail will be C/20, and Fig. 2 shows both the Experimental change in neutron transmission (Fig. 2a) and simulated changes in total Li^+^ concentration (Figs. 2b–2d) at that rate for comparison. The separate electrolyte and solid phase concentration profiles can be found in Supporting Information, Fig. S4. The Experimental changes in neutron transmission and simulated changes in electrolyte, solid, and total Li^+^ concentration can also be found in the Supporting Information for the other discharge rates of C/10 (Fig. S5), C/5 (Fig. S6), and C/2.5 (Fig. S7). Note that in all cases the x-axis was the thickness between the current collectors on a relative scale from 0 (LCO current collector) to 1 (LTO current collector), where the midpoint of the separator in the cell was located at 0.406.

#### Experimental changes in neutron transmission.—

The *x*-axis in Fig. 2 corresponds to the LCO current collector at “0”, the LCO near the separator at “0.397,” the LTO near the separator at “0.415,” and the LTO current collector at “1”. Within the LCO electrode at a discharge rate of C/20, the changes in neutron transmission suggested a relatively uniform lithiation throughout the electrode thickness as the discharge proceeded. The transmission through the LCO electrode gradually decreased as the discharge proceeded (Fig. 2a), which would be expected as the lithium intercalates within the LCO particles.^5,7^ The relatively flat profile suggested the electrode fairly uniformly contributed to the electrochemical reaction throughout its thickness. The deviations from a relatively flat profile near the separator and current collector would be expected because within those regions the Li^+^ concentration cannot change (at all at the current collector, and only a relatively small amount within the electrolyte phase within the separator region).

The LTO electrode overall has an increase in relative neutron transmission throughout its thickness (Fig. 2a), which would be expected due to the delithiation of the LTO during discharge. However, the changes in transmission were not as uniform throughout the electrode thickness. There were significantly greater increases in the neutron transmission in the LTO regions closer to the separator region, and a noticeable front of increasing neutron transmission which propagated towards the current collector as the discharge process proceeded. This outcome has previously been discussed, and the nonuniform neutron transmission and delithiation has been attributed to the Li^+^ transport limitations in the very thick electrodes. These transport limitations become more restrictive at higher discharge rates/current densities, and thus as the discharge rate increased the changes in relative neutron transmission (and equivalently Li^+^ concentration) become increasingly localized to near the separator region for both electrodes (see Supporting Information, Figs. S5–S7).

#### Fixed conductivity simulation of Li^+^ concentration changes.—

At C/20 discharge rate, the lithiation of the LCO during discharge was fairly uniform across the electrode thickness (Fig. 2b), consistent with the changes in neutron transmission (Fig. 2a). There was a slight gradient of higher lithiation near the separator region at some extents of discharge (e.g., 75% discharge capacity) which likely reflected the lower concentration polarization within this region, but generally the concentration profiles were relatively uniform compared to the LTO. In contrast, within the LTO electrode, delithiation preferentially initiated near the edges of the electrode at both the current collector and separator at the beginning of discharge, with initially greater delithiation near the separator. This phenomena has been previously observed and has been attributed to resulting from relatively low electronic polarization near the current collector and ionic polarization near the separator.^7^ This simulated behavior for changes in total Li^+^ concentration was qualitatively in conflict with the experimental outcomes, and thus Fixed Conductivity was the case with the greatest deviation from the experimental outcomes both with regards to the discharge curve profile and the changes in Li^+^ concentration during cell discharge.

#### Variable conductivity simulation of Li^+^ concentration changes.—

At C/20 discharge rate, for the Variable Conductivity case LCO lithiation was also fairly uniform across the electrode thickness as the discharge proceeded (Fig. 2c), though with slight gradients of increased lithiation near the separator region. This outcome was consistent with both the Experimental changes in neutron transmission and the Fixed Conductivity cases (Figs. 2a, 2b). On the LTO side of the cell, the Variable Conductivity simulation resulted in a propagation front of delithiation within the electrode, where near the separator the LTO reached a maximum delithiation first and then there was a relatively sharp gradient in Li^+^ concentration in the LTO electrode which propagated towards the current collector as the discharge proceeded. Note that the absolute change in Li^+^ concentration near the separator reached a maximum very quickly (within the first 25% of the discharge) and then this delithiation propagated towards the current collector. While the propagation of the delithiation front in the LTO was consistent with the neutron transmission Experimental system (Fig. 2a), the changes in neutron transmission near the separator did not level out at a maximum value as quickly as the Li^+^ concentration reached a minimum in the Variable Conductivity simulation. Also, at the end of the discharge the Variable Conductivity case (and also the Fixed conductivity case) ended with a nearly uniform change in Li^+^ concentration throughout the LTO electrode thickness. This outcome was inconsistent with the neutron transmission data, which still had noticeable variation for approximately half of the electrode thickness moving towards the current collector even at the conclusion of the discharge. Another discrepancy between the Experimental neutron transmission changes and the Variable Conductivity simulation was the relative magnitude of the changes within the LTO and LCO electrodes. The simulated total change in Li^+^ concentration in the LCO electrode at the end of discharge was slightly greater than 13 mol l^−1^, while the change in the LTO electrode was at most 8.4 mol l^−1^. However, the Experimental changes in neutron transmission suggested that the LTO electrode experienced greater changes in Li^+^ concentration (with corresponding greater changes in relative transmission).

An additional insight from the Li^+^ concentration profiles from the simulations was the cause of a change where the Fixed Conductivity switched to having a higher discharge voltage than the Variable Conductivity discharge curve in some situations. Close inspection of Figs. 1a and 1b revealed that initially the Variable Conductivity had a higher discharge voltage, but that at ∼540 min for C/20, at ∼270 min for C/10, and at ∼130 min for C/5 the Fixed Conductivity discharge profile had a higher voltage. This outcome resulted from differences in the Li^+^ concentration distribution in the electrolyte phase. For the Fixed Conductivity, as discussed earlier, the delithiation of the LTO electrode initiated from the two edges of the electrode near the separator and the current collector (though slightly more so near the separator), and then propagated towards the center of the electrode. However, for the Variable Conductivity Case, towards the end of discharge, the delithiation near the separator was complete and electrochemical activity was focused near the current collector, where the polarization due to Li^+^ transport was very high. The Li^+^ electrolyte concentration at the end of discharge has a gradient that propagates much deeper into the LTO electrode in the Variable Conductivity case as opposed to the Fixed Conductivity case, consistent with the greater polarization experienced at later discharge times for the Variable Conductivity simulation (see Supporting Information, Fig. S4a for the Li^+^ electrolyte concentration at the end of discharge for both cases).

#### Initial gradient simulation of Li^+^ concentration changes.—

Qualitatively, at C/20 discharge rate, the resulting profile for change in Li^+^ concentration for the Initial Gradient case in Fig. 2d has the best match with the Experimental changes in neutron transmission (Fig. 2a) relative to the former two cases. After 25% of the discharge time, approximately half of the final change in Li^+^ concentration change near the separator side for the LTO electrode has occurred, which was consistent with the approximate change in the relative neutron transmission in the Experimental profile at the same location and time. The qualitative propagation of the Li^+^ gradient towards the current collector was consistent for both the Initial Gradient and Experimental profiles. At the end of discharge, there were regions of the LTO electrode near the current collector that have largely remained unchanged in Li^+^ concentration, which was qualitatively the best match with the Experimental neutron transmission profile. Finally, the total change in Li^+^ concentration in the LTO electrode near the separator at the end of discharge were greater than the changes in the LCO electrode near the separator, which was consistent with the greater total relative change observed in the LTO near the separator compared to LCO near the separator in the Experimental neutron transmission profiles. Note that the simulation resulted in a higher capacity than the Experimental case, and thus the Initial Gradient had an overall change in Li^+^ concentration in the LTO electrode which was greater than the Experimental case. Thus, the Initial Gradient case resulted in Li^+^ concentration profiles which were most consistent with the Experimental neutron transmission profiles.

It is noted that with high enough resolution and alignment, an approximate plateau region with very little change in neutron transmission would be expected at the depths corresponding to the separator location, because there was no solid phase in this 25 *μ*m region and the Li^+^ concentration change in the electrolyte phase was calculated to be relatively small. Even though the pixel pitch for the neutron images was 6.5 *μ*m,^7^ there were two factors that limited observing a plateau in the separator region. First, it was difficult to obtain truly perfect alignment of the cell. The combined thickness of the region for the two electrodes and separator as determined from neutron images was 5% greater than the combined thicknesses measured using a micrometer, and this difference was attributed to a slight tilt of the cell. The misalignment would result in slight contributions from the edges of both the LCO and LTO electrodes that extended into the separator region. Even with perfect alignment, there would be some signal from the electrode edges contributed to the nearest pixels in the separator region, meaning that at perfect alignment only one or two pixels would experience the minimal intensity change, and would make observing a plateau region challenging.

While thick and sintered electrodes without conductive additives in particular were necessary for some of the behavior described in this report, it is expected that similar outcomes would occur with materials beyond LTO and LCO. A number of Li-ion active materials have been reported to have electronic conductivity which was sensitive to the extent of lithiation, particularly transition metal oxides.^28,29^ Such materials, if incorporated into a sintered electrode framework, would need to have lithiation-dependent electronic conductivity to simulate their electrochemical cell properties. The initial gradient in lithiation state through the electrode thickness resulting from cell charging is expected to be a more general consequence of thick electrodes, and should be more broadly taken into consideration when designing thick electrode cells, in particular those with sintered active material architectures.

### Charging process.—

Before the neutron imaging measurements, the experimental cells underwent charge/discharge cycling to confirm they were functioning properly and then arrived at the beamline in the charged state. Thus, neutron data was not collected during the initial charge cycle for any cell. However, neutron images were collected throughout the process of the cell being subsequently charged/discharged at the beamline and thus there was charging information (both charging voltage curves and neutron imaging data) collected at a rate of C/20. The measured changes in neutron transmission relative to the initiation of charge and charging profile can be found in the Supporting Information, Figs. S8 and S9. This C/ 20 charge cycle was after multiple test charge/discharge cycles, and immediately following a C/20 discharge cycle.

For comparison with the experimental charging data, charging the cell was simulated using the same conditions as the Initial Gradient case. The charge cycle was simulated following an initial charge at C/20 and discharge at C/20 (e.g., the “second charge cycle” was simulated). The experimental and simulated second charge cycle voltage profiles had more offset in polarization and total capacity compared to the C/20 discharge profiles. However, note that the optimization for the initial state of charge for the simulated first charge cycle was based on minimizing the error of the first discharge at C/20, thus more error would be expected for a subsequent charge cycle. The simulated time for the second charge cycle was ∼793 min, and the experimental charging time used for comparison was slightly more than 800 min. The LCO on charge had more of a gradient in the neutron transmission data relative to the model, particularly at later times in the charge. At early times of charge, experimentally the neutron transmission data suggested more lithiation in the LTO near the current collector than was accounted for in the simulation. The general trend of increasing lithiation in the LTO near the separator as a function of extent of charge and a propagation towards the current collector was still observed in both the experimental data and the model.

The behavior of the experimental cell was generally captured by the Initial Gradient simulation case, although the observed lithiation/ delithiation behavior and voltage profiles were quantitatively not as well matched as the discharge process. It is difficult to diagnose the cause of the discrepancies without having neutron transmission information from the very first cycle(s), which would allow capturing the full cell history to compare to simulations. Also, for the voltage profiles the second simulated charge being consistent with the first simulated charge was expected given the same cell conditions were being modeled and that the modeled discharge coulombic efficiency was high (e.g., returned to approximately the same initial state before the second charge). It is speculated that the differences between the experimental profile and simulated profile resulted from processes not included in the model. One such process is capacity fading, which will shift the polarization curves due to different net lithiation states in the electrodes during charge and discharge and will change the maximum total capacity which can be achieved. *In operando* neutron imaging experiments on newly prepared LTO/LCO sintered cells would improve understanding the source of the differences and provide a more comprehensive cell history for comparison, and thus such experiments will be planned for future investigations.

### Possible further considerations.—

The Initial Gradient simulation which incorporated both a matrix electronic conductivity which was dependent on the extent of lithiation for multiple points within each electrode and included an initial gradient in Li^+^ concentration due to the charging process of the cell was the best match to the Experimental polarization curves and changes in neutron transmission. However, the model of the system still has areas for future improvement. There was generally a mismatch in final delivered capacity at C/10 and C/5, where the simulations for the best cases had good match with the Experimental polarization curve at earlier discharge times, but then overestimated the discharge time/capacity. It is suspected that this may arise from difficulty in matching the detailed cell history and lithiation profile, and this lack of the full cell history for comparison was suspected as also impacting the simulations of the charging process. The cells go through multiple charge/ discharge cycles for quality control purposes before neutron imaging experiments, and then have to rest and be slightly recharged due to shipment to the neutron user facility. Replicating these detailed cell conditions precisely would be challenging (and would need to account for other factors such as first cycle irreversible capacity loss) and thus without knowing the precise state of charge as a function of location in the cell exact matching of the experimental capacity available is challenging. There is also potential two- and three-dimensional heterogeneity in the cell (e.g., the pore sizes and connectivity) which cannot be accounted for in the 1-D simulation model. However, to appropriately account for this heterogeneity in simulations much more information would be needed, including detailed 3-D pore reconstructions.

Another simplification in the models was ignoring the contributions of resistance from particle-particle surface contacts. The conductivity in the Variable Conductivity and Initial Gradient cases were dependent on lithiation, but the bulk material electronic conductivity values were used. These did not include then the resistance associated with conduction between particle contacts, and thus the electronic conductivity for these cases is expected to be an overestimation. In any case, a detailed experimental assessment of the electronic conductivity of sintered electrodes will need to be conducted to have further confidence in the assumption of electronic conduction through the active material and the particle-particle connections.

## Conclusions

This report described incorporation of variable matrix electronic conductivity and an initial lithiation gradient within the electroactive material as a function of electrode depth during simulation of the discharge of sintered thick electrodes. The match of the simulations with discharge polarization curves was improved when incorporating the updated model. Further support for incorporation of these properties in the model was given via comparison of calculated lithium concentration profiles to *in operando* neutron transmission data, where in particular the initial gradient in lithiation in the electrodes due to the charging cycle was needed to capture the correct qualitative behavior of the lithiation propagation within the anode and the relative extents of changes in lithium concentration within the anode and cathode near the separator within the cell. It is expected that improvements in modeling of sintered electrodes will aid in understanding their performance limits as a function of different factors which can be influenced by processing (e.g., thickness and porosity) and in the selection of alternative materials for this high energy density system at the cell level.

## Supplementary Material

supplementary material

## Figures and Tables

**Table I.** Electrode parameters used in simulation.

Parameters	Cathode value	Anode value

Thickness (*μ*m)	468, Measured	691, Measured
Solid State Li^+^ Diffusivity (m^2^ s^−1^)	3.5 × 10^−13^ ^14^	2.0 × 10^−12^ ^15^
Active Material Radius (m)	2 × 10^−7^ ^16^	1.7 × 10^−7^ ^17^
Porosity	0.38, Measured	0.42, Measured
Bruggeman Exponent	1.5	1.5
Rate Constant (m^2.5^ mol^−0.5^ s^−1^)	3.10 × 10^−13^ ^18^	3.90 × 10^−13^ ^19^
Density (kg m^−3^)	5010^20^	3480^21^
Capacity (mA h g^−1^)	274^22^	175^22^
Fixed conductivity (S m^−1^)	0.5	0.5
Variable conductivity (S m^−1^)	7000 × (1−x)^2^ + 5 × (1−x) + 0.054, 0.5 ⪬ x ⪬ 1.0, in Li_x_CoO_2^10,11^_	Exp(4.37 × (1 − y)^200^) × 300 × (y + 10^−6^)^0.38^ × 5^(y − 1)^, 0 ⪬ y ⪬ 1.0, in Li_4+3y_Ti_5_O_12^12^_
Open Circuit Voltage (V)	0.076 × tanh(64.13–51.30x) + 1.50 × tanh(50.85 × 51.71x) + tanh(9.09–21.09x) + 0.21 × tanh(3.47–5.83x) + 0.25 × tanh(8.10x + 4.29) − 0.022 × tanh(1.06x − 0.52) × 2.61, 0.5 ⩽ x ⩽ 1.0, in Li_x_CoO_2_^15^	0.21 × Exp(−116.96y) + 0.45 × Exp(−5000y) + 0.27706 × Exp(−1010.1y) + 1.54 − Exp(50(y − 0.87)) × 0.001, 0 ⩽ y ⩽ 1.0, in Li_4+3y_Ti_5_O_12_, Fitted from Experimental

**Table II.** Electrolyte and other parameters used in simulation.

Electrolyte and Other Parameters	Value

Transference number, $t_{+}^{0}$	0.415^23^
Initial Concentration (mol m^−3^)	1200, Experimental
Thermodynamic Factor, $\left(1+\frac{\partial lnf\pm }{\partial lnc}\right)$ (1 – $t_{+}^{0}$)	0.28687 c^2^ + 0.74678 c + 0.44103^24^
Conductivity (S m^−1^)	0.1297c^3^ + 2.51c^1.5^ + 3.329c^24^
Diffusivity (m^2^ s^−1^)	(−6.9444c^2^ + 7.3611c + 2.65) × 10^−10^, c < 0.8, 6.4753 × Exp(−0.573c) × 10^−10^, c ⩾ 0.8^24^
Temperature (K)	298.15, Room Temperature
Gas Constant (J K^−1^ mol^−1^)	8.3145
Faraday Constant (A s mol^−1^)	96485
Separator Thickness (*μ*m)	25, Manufacturer
Separator Bruggeman Exponent	2.2^25^
Internal resistance (Ω m^2^)	0.0034, Estimated from Experimental

## References

[1] Etacheri V, Marom R, Elazari R, Salitra G, and Aurbach D, Energy Environ. Sci, 4, 3243 (2011).

[2] Gallagher KG , J. Electrochem. Soc, 163, A138 (2016).

[3] Lai W, Erdonmez CK, Marinis TF, Bjune CK, Dudney NJ, Xu F, Wartena R, and Chiang YM, Adv. Mater, 22, 139 (2010). 20301129 10.1002/adma.200903650

[4] Sotomayor ME, Torre-Gamarra CDL, Levenfeld B, Sanchez JY, Varez A, Kim GT, Varzi A, and Passerini S, J. Power Sources, 437, 226923 (2019).

[5] Nie Z, McCormack P, Bilheux HZ, Bilheux JC, Robinson JP, Nanda J, and Koenig GM, J. Power Sources, 419, 127 (2019).

[6] Robinson JP, Ruppert JJ, Dong H, and Koenig GM, J. Appl. Electrochem, 48, 1297 (2018).

[7] Nie Z, Ong S, Hussey DS, LaManna JM, Jacobson DL, and Koenig GM, Mol. Syst. Des. Eng, 5, 245 (2020). 10.1039/c9me00084d PMC8739910 35003760

[8] Doyle M and Newman J, J. Electrochem. Soc, 143, 1890 (1996).

[9] Fuller TF, Doyle M, and Newman J, J. Electrochem. Soc, 141, 1 (1994).

[10] Saadoune I and Delmas C, J. Mater. Chem, 9, 1135 (1999).

[11] Levasseur S, Ménétrier M, Suard E, and Delmas C, Solid State Ionics, 128, 11 (2000).

[12] Young D, Ransil A, Amin R, Li Z, and Chiang YM, Adv. Energy Mater, 3, 1125 (2013).

[13] Martínez-Rosas E, Vasquez-Medrano R, and Flores-Tlacuahuac A, Comput. Chem. Eng, 35, 1937 (2011).

[14] Xie J, Imanishi N, Matsumura T, Hirano A, Takeda Y, and Yamamoto O, Solid State Ionics, 179, 362 (2008).

[15] Zaghib K, Simoneau M, Armand M, and Gauthier M, J. Power Sources, 81–82, 300 (1999).

[16] Qi Z and Koenig GM, ChemistrySelect, 1, 3992 (2016).

[17] Qi Z and Koenig GM, J. Power Sources, 323, 97 (2016).

[18] Habte BT and Jiang F, Microporous Mesoporous Mater, 268, 69 (2018).

[19] Chen J, Yang L, Fang S, Hirano SI, and Tachibana K, J. Power Sources, 200, 59 (2012).

[20] Mao J, Tiedemann W, and Newman J, ECS Trans, 58, 71 (2014).

[21] Kataoka K, Takahashi Y, Kijima N, Akimoto J, and Ohshima KI, J. Phys. Chem. Solids, 69, 1454 (2008).

[22] Nitta N, Wu F, Lee JT, and Yushin G, Mater. Today, 18, 252 (2015).

[23] Capiglia C, Saito Y, Kageyama H, Mustarelli P, Iwamoto T, Tabuchi T, and Tukamoto H, J. Power Sources, 81–82, 859 (1999).

[24] Nyman A, Behm M, and Lindbergh G, Electrochim. Acta, 53, 6356 (2008).

[25] Finegan DP, Cooper SJ, Tjaden B, Taiwo OO, Gelb J, Hinds G, Brett DJL, and Shearing PR, J. Power Sources, 333, 184 (2016).

[26] Delattre B, Amin R, Sander J, Coninck JD, Tomsia AP, and Chiang YM, J. Electrochem. Soc, 165, A388 (2018).

[27] Zhou H, An K, Allu S, Pannala S, Li J, Bilheux HZ, Martha SK, and Nanda J, ACS Energy Lett, 1, 981 (2016).

[28] Levi MD, Lu Z, Gofer T, Cohen Y, Cohen Y, Aurbach D, Vieil E, and Serose J, J. Electroanal. Chem, 479, 12 (1999).

[29] Griffith KJ, Wiaderek KM, Cibin G, Marbella LE, and Grey CP, Nature, 559, 556 (2018). 30046074 10.1038/s41586-018-0347-0

