# Supplementary material for: Thick Sintered Electrode Lithium-Ion Battery Discharge Simulations: Incorporating Lithiation-Dependent Electronic Conductivity and Lithiation Gradient Due to Charge Cycle
Source: J Electrochem Soc. Author manuscript; Available in PMC 2021 Dec 6. (PMC8647443; doi:10.1149/1945-7111/abc747)
Supplement: supplementary material [file NIHMS1753166-supplement-supplementary_material.docx]

**Supporting information**

**Title:** Pore Alignment Impacts on Lithium Ion Transport and Rate Capability of Thick Sintered Electrodes

**Authors names and affiliations**:

Ziyang Nie,^a^ Rohan Parai,^b^ Chen Cai,^a^ Charles Michaelis,^a^ Jacob M. LaManna,^c^ Daniel S. Hussey,^c^ David L. Jacobson,^c^ Dipankar Ghosh,^b^ Gary M. Koenig Jr.*^a^

^a^ Department of Chemical Engineering, University of Virginia, 102 Engineers Way, Charlottesville, VA, USA 22904-4741

^b^ Department of Mechanical and Aerospace Engineering, Old Dominion University, Norfolk, VA, USA 23529

^c^ National Institute of Standards and Technology Physical Measurements Laboratory, Gaithersburg, MD, USA 20899-8461

**Corresponding author:**

Gary M. Koenig Jr.

[gary.koenig@virginia.edu](mailto:gary.koenig@virginia.edu)

**Neutron imaging experiment set up and data analysis.**

Neutron imaging experiments were carried out at the thermal neutron imaging beamline at the National Institute for Standards and Technology (NIST) Center for Neutron Research.^[[1]](#endnote-1)^ The experimental setup is shown in Fig. S1a. The cell was held with a clip in front of the detector and two stainless steel foils were used as leads connecting the positive and negative electrode of the cell. The detector was an Andor Neo sCMOS camera with a 1:1 macro lens that gives a pixel pitch of 6.5 micrometer. A 20-micrometer thick gadolinium oxysulfide scintillator was used. After starting the experiment, radiographs were collected every minute. An example of a raw radiograph collected is shown in Fig. S1b. While analyzing the data, three radiographs were averaged for each time point and combined in order to reduce signal noise. To compare neutron imaging results to simulated Li^+^ concentration throughout the thickness of the cell during discharge, radiographs of all time points were normalized relative to the image that combined several radiographs taken before cycling (the “no current” image) and a dark field correction was carried out at the same time. The change in the transmission intensity of each pixel relative to the “no current” image (ΔTransmission) were shown using a color scale in this manuscript (Fig. S1c). Since ^6^Li had the largest neutron cross section than other species (except ^1^H) in the system and the concentration of Li was higher (and had the greatest change in concentration) than other elements in the cell regions between the current collectors, changes in Li^+^ (in particular in the solid phase within the electrode regions) should result in most of the observed changes in neutron transmission.^[[2]](#endnote-2)^ In the normalized color scale images (Fig. S1c), the regions of lower transmission (higher Li^+^ concentration) relative to the initial state are shown in blue and the regions of relatively higher transmission (lower Li^+^ concentration) compared to the initial state are shown in red. To highlight the contrast between anode and cathode, the image displayed Fig. S1c was taken at the end of the C/10 discharge of the LTO_ICE_/LCO cell. In Fig. S1c, the blue region represents LCO electrode which had higher Li^+^ concentration than the initial state at the end of discharge and the red region represents LTO electrode which had lower Li^+^ concentration at the end of discharge relative to the initial state.

To quantitatively analyze the transmission radiographs, a 1000-pixel wide line scan was used across the electrode area from bottom to the top (z-direction in Fig. S1c). Before being transported to NIST, the cells used in the neutron imaging were charged to 2.7 V at a rate of C/20. Then, after the cells were aligned for experiments at NIST (Fig. S1a), they were charged again at a rate of C/20 to 2.7 V to compensate for any capacity lost between their previous charge cycle and arriving at NIST.


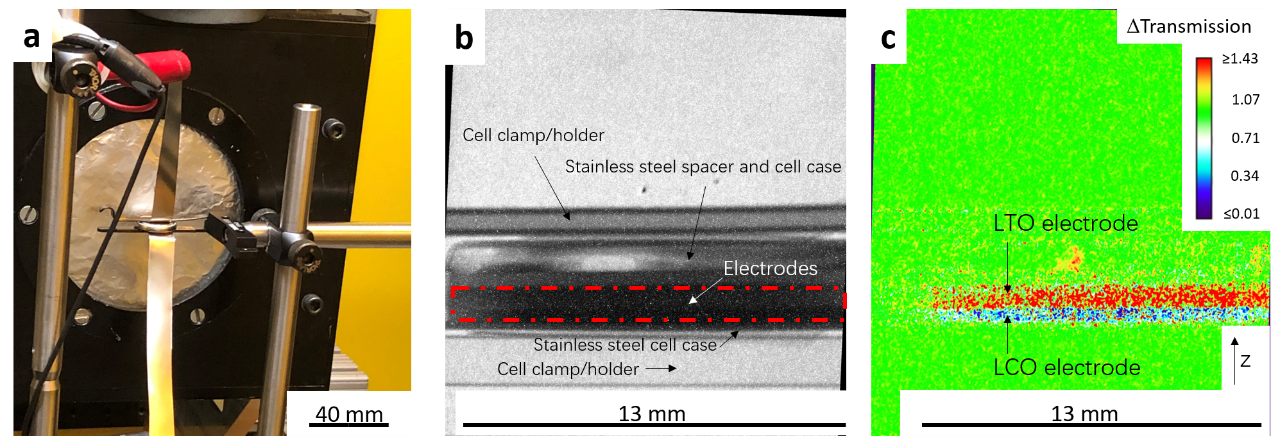


**Fig. S1** (a) Photograph of experimental setup used for neutron imaging. (b) Example of a raw radiograph image of the coin cell region. (c) Example of the change in transmission for a radiograph of the cell after normalizing relative to the “no current” image. A color scale was used to show the relative change in neutron transmission. The black arrow to the bottom right of the image depicts the z-direction used for line scan analysis of the cell (thickness/depth dimension). Note that the brightest red regions have ΔT ≥ 1.43 and deepest blue regions have ΔT ≤ 0.01 and do not reflect the absolute maximum or minimum ΔT values and the same color scale was used for all neutron images displayed in this work.


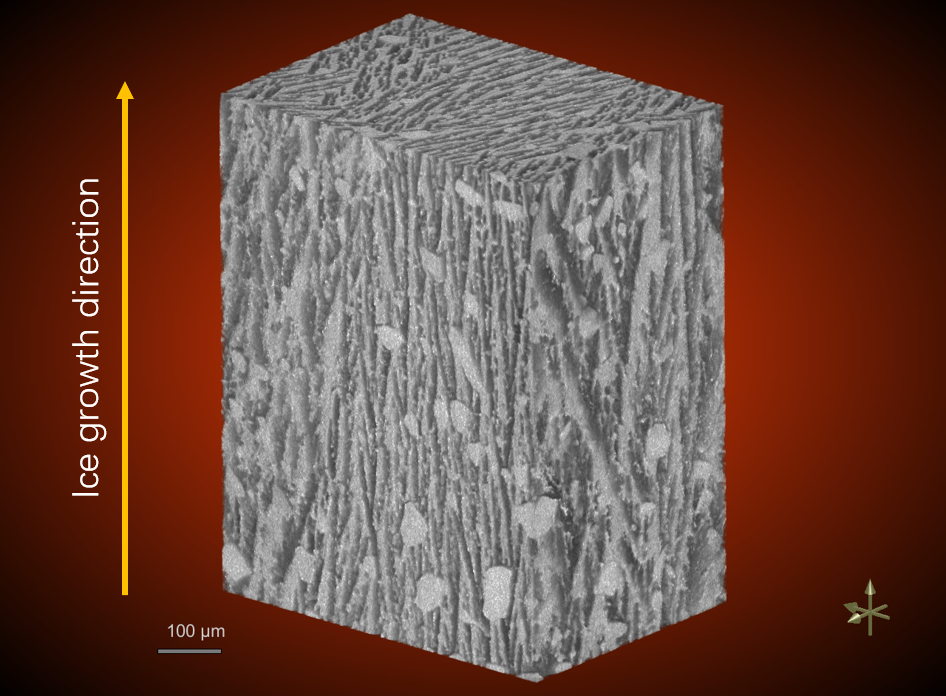


**Fig. S2** X-CT image of a sample region for a LTO_ICE_ pellet.


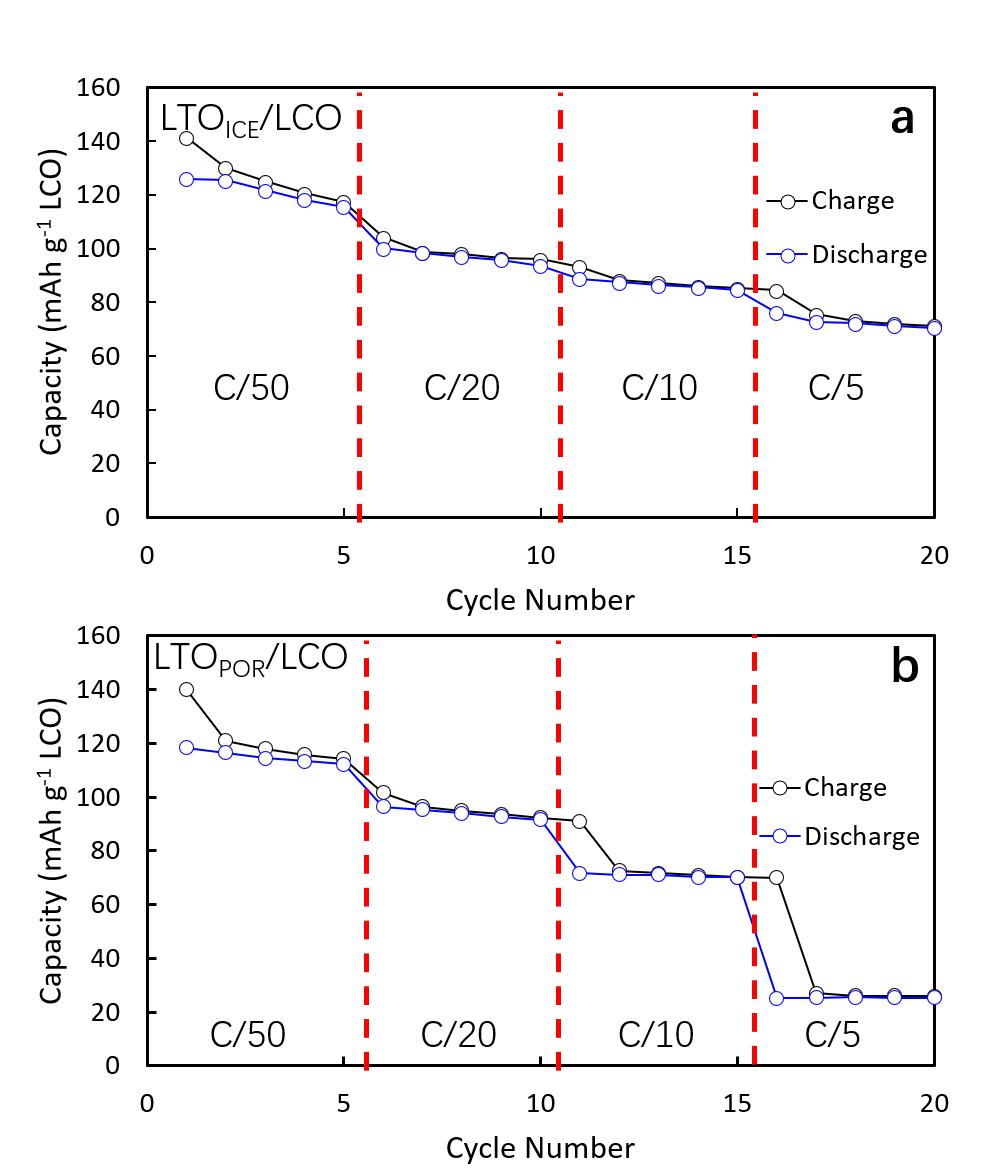


**Fig. S3** Rate capability test of a representative (a) LTO_ICE_/LCO and (b) LTO_POR_/LCO cell. The discharge rate for each cycle is noted in the figure. For both cells, the charge rate for the first 5 cycles (C/50 discharge) was C/50. The charge rate for all following cycles was C/20.


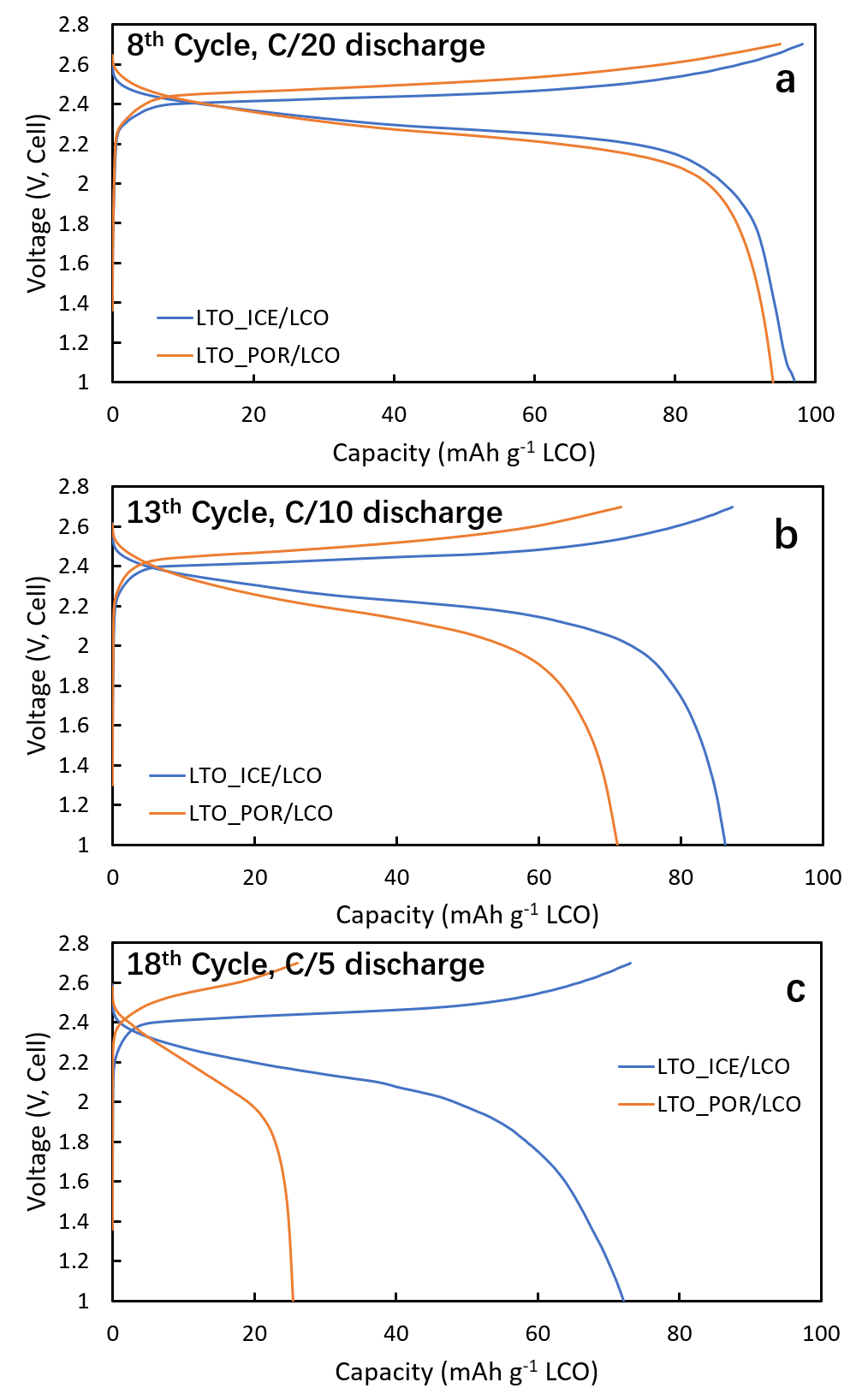


**Fig. S4** Charge/discharge profiles corresponding to the (a) 8^th^ cycle, (b) 13^th^ cycle, and (c) 18^th^ cycle from Fig. S3. The profiles correspond to the LTO_ICE_/LCO (blue) and LTO_POR_/LCO (orange) cells. For all cases, the charge was at C/20. The discharge was conducted at (a) C/20, (b) C/10, and (c) C/5.

**Table S1**. Parameters used in calculations

| Parameters | LTO_ICE_/LCO | LTO_POR_/LCO | LTO_ICE2_/LCO | Source |
| --- | --- | --- | --- | --- |
| Thickness of negative electrode/LTO (m) | 9.10×10^-4^ | 8.95×10^-4^ | 8.56×10^-4^ | Measured |
| Thickness of separator (m) | 5.0×10^-5^ | | | Manufacturer |
| Thickness of positive electrode/LCO (m) | 4.57×10^-4^ | 4.64×10^-4^ | 4.57×10^-4^ | Measured |
| Bulk LiPF_6_ concentration (mol m^-3^) | 1200 | | | Manufacturer |
| Solid-state Li^+^ diffusion coef. in anode (m^2^ s^-1^) | 2×10^-12^ | | | Ref. ^[[3]](#endnote-3)^ |
| Solid-state Li^+^ diffusion coef. in cathode (m^2^ s^-1^) | 3.5×10^-13^ | | | Ref. ^[[4]](#endnote-4)^ |
| Radius of anode active particles (m) | 1.7×10^-7^ | | | Ref. ^[[5]](#endnote-5)^ |
| Radius of cathode active particles (m) | 2.0×10^-7^ | | | Ref. ^[[6]](#endnote-6)^ |
| Volume fraction of electrolyte in negative electrode | 0.57 | 0.56 | 0.57 | Based on measured porosity. |
| Volume fraction of electrolyte in separator | 0.39 | | | Manufacturer |
| Volume fraction of electrolyte in positive electrode | 0.36 | 0.36 | 0.37 | Based on measured porosity. |
| Conductivity of negative matrix  (S m^-1^) | 7000 × (1 – x)2 + 5 × (1 – x) + 0.054,  0.5≤x≤1.0 in Li_x_CoO_2_ | | | Ref. ^[[7]](#endnote-7)^,^[[8]](#endnote-8)^ |
| Conductivity of positive matrix  (S m^-1^) | Exp(4.37× (1 - y)200) ×300 × (y + 10-6)0.38 × 5(y - 1),  0≤y≤1.0 in Li_4+3y_Ti_5_O_12_ | | | Ref. ^[[9]](#endnote-9)^ |
| Coulombic gravimetric capacity of negative material (mAh g^-1^) | 175 | | | Ref. ^[[10]](#endnote-10)^ |
| Coulombic gravimetric capacity of positive material (mAh g^-1^) | 274 | | | Ref. 10 |
| Density of negative insertion material (kg m^-3^) | 3480 | | | Theoretical crystal density, Ref ^[[11]](#endnote-11)^ |
| Density of positive insertion material (kg m^-3^) | 5010 | | | Theoretical crystal density. Ref. ^[[12]](#endnote-12)^ |
| Rate constant for negative reaction (m^2.5^ mol^-0.5^ s^-1^) | 3.10 × 10^-13^ | | | Ref. ^[[13]](#endnote-13)^ |
| Rate constant for positive reaction (m^2.5^ mol^-0.5^ s^-1^) | 3.90 × 10^-13^ | | | Ref. ^[[14]](#endnote-14)^ |
| Bruggeman exponent α for cathode | 1.5 | | | Value for randomly packed spheres |
| Bruggeman exponent α for anode | 1.0 | 1.5 | 1.0 | Value for perfectly aligned pores (1.0) and randomly packed spheres (1.5) |
| Separator Bruggeman Exponent | 2.5 | | | Ref. ^[[15]](#endnote-15)^ |
| Open circuit voltage for anode (V) | 0.21 × Exp(-116.96y) + 0.45 × Exp(-5000y) + 0.27706 ×  Exp(-1010.1y) + 1.54, 0 ≤ y ≤ 1.0 in Li_4+3y_Ti_5_O_12_ | | | Fitted from experimental data |
| Open circuit voltage for cathode (V) | 0.076 × tanh(64.13 –51.30x) + 1.50 × tanh(50.85× 51.71x) + tanh(9.09 –21.09x) + 0.21 × tanh(3.47– 5.83x) + 0.25 ×tanh(8.10x + 4.29) – 0.022× tanh(1.06x – 0.52) + 2.61,  0.5 ≤ x ≤ 1.0 in Li_x_CoO_2_ | | | Ref. 12 |
| Electrolyte transference number | 0.415 | | | Ref. ^[[16]](#endnote-16)^ |
| Thermodynamic Factor, $(1+\frac{\partial lnf_{\pm}}{\partial lnc})(1-t_{+}^{0})$ | 0.28687 c^2^ + 0.74678 c + 0.44103 | | | Ref. ^[[17]](#endnote-17)^ |
| Electrolyte conductivity (S m^-1^) | 0.1297c^3^ + 2.51c^1.5^ + 3.329c | | | Ref. 17 |
| Electrolyte diffusivity (m^2^ s^-1^) | (-6.9444c^2^ + 7.3611c + 2.65) × 10^-10^, c < 0.8,  6.4753 × Exp(-0.573c) × 10^-10^, c ≥ 0.8 | | | Ref. 17 |
| Temperature (K) | 298.15 | | | Room temperature |
| Internal resistance (Ω m^2^) | 0.0034 | | | Estimated from experimental data |


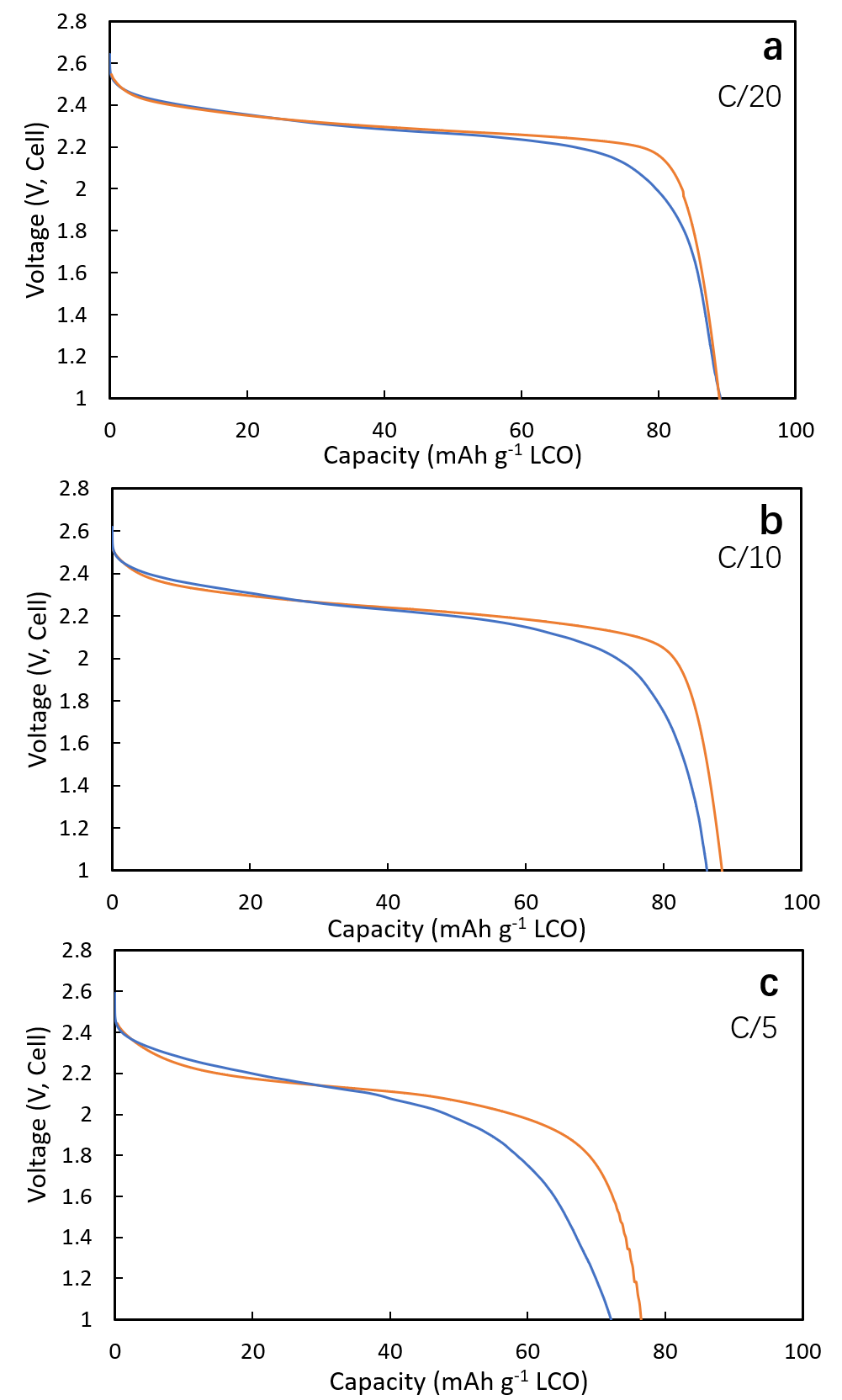


**Fig. S5** Experimental (blue) and calculated (orange) discharge profiles at (a) C/20, (b) C/10 and (c) C/5 for LTO_ICE_/LCO cell.


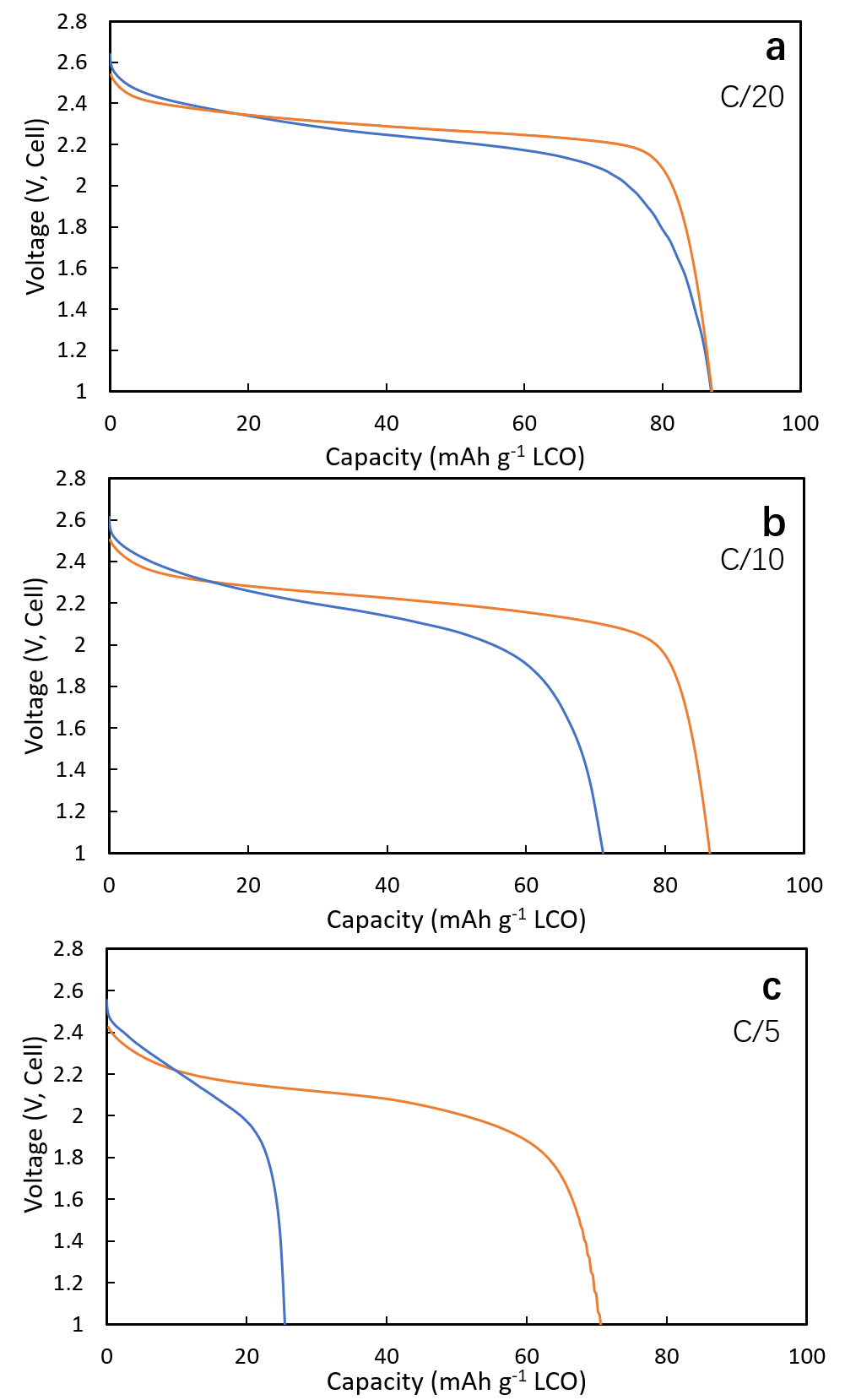


**Fig. S6** Experimental (blue) and calculated (orange) discharge profiles at (a) C/20, (b) C/10 and (c) C/5 for LTO_POR_/LCO cell.


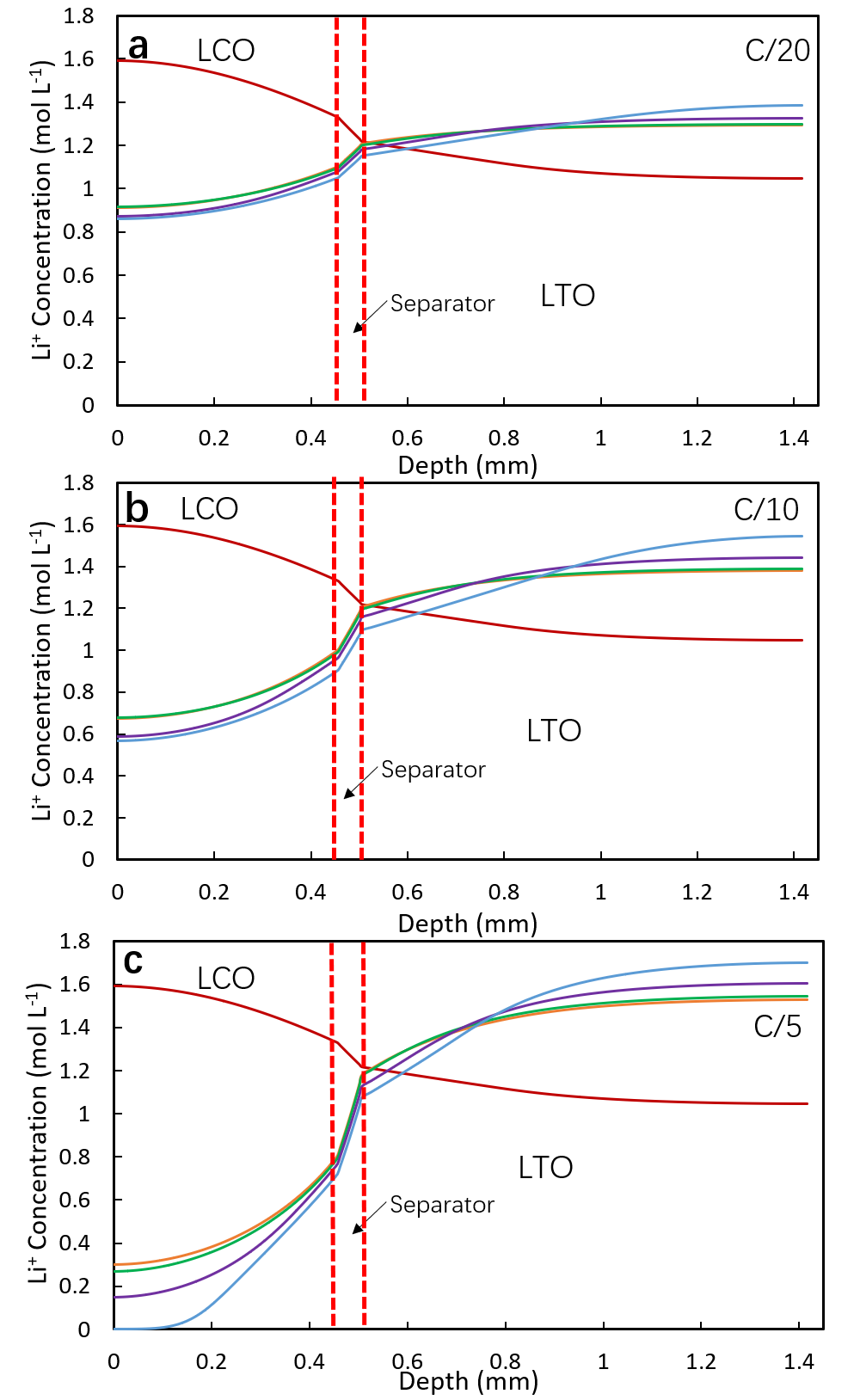


**Fig. S7** Concentration profiles in electrolyte phase for LTO_ICE_/LCO cell at (a) C/20, (b) C/10 and (c) C/5. The different curves correspond to the different extents of discharge capacity delivered, with the concentrations being shown for 0 % (magenta), 25 % (orange), 50 % (green), 75 % (purple), and 100 % (blue). The profile for 0 % capacity is right at the conclusion of the C/20 charge process.


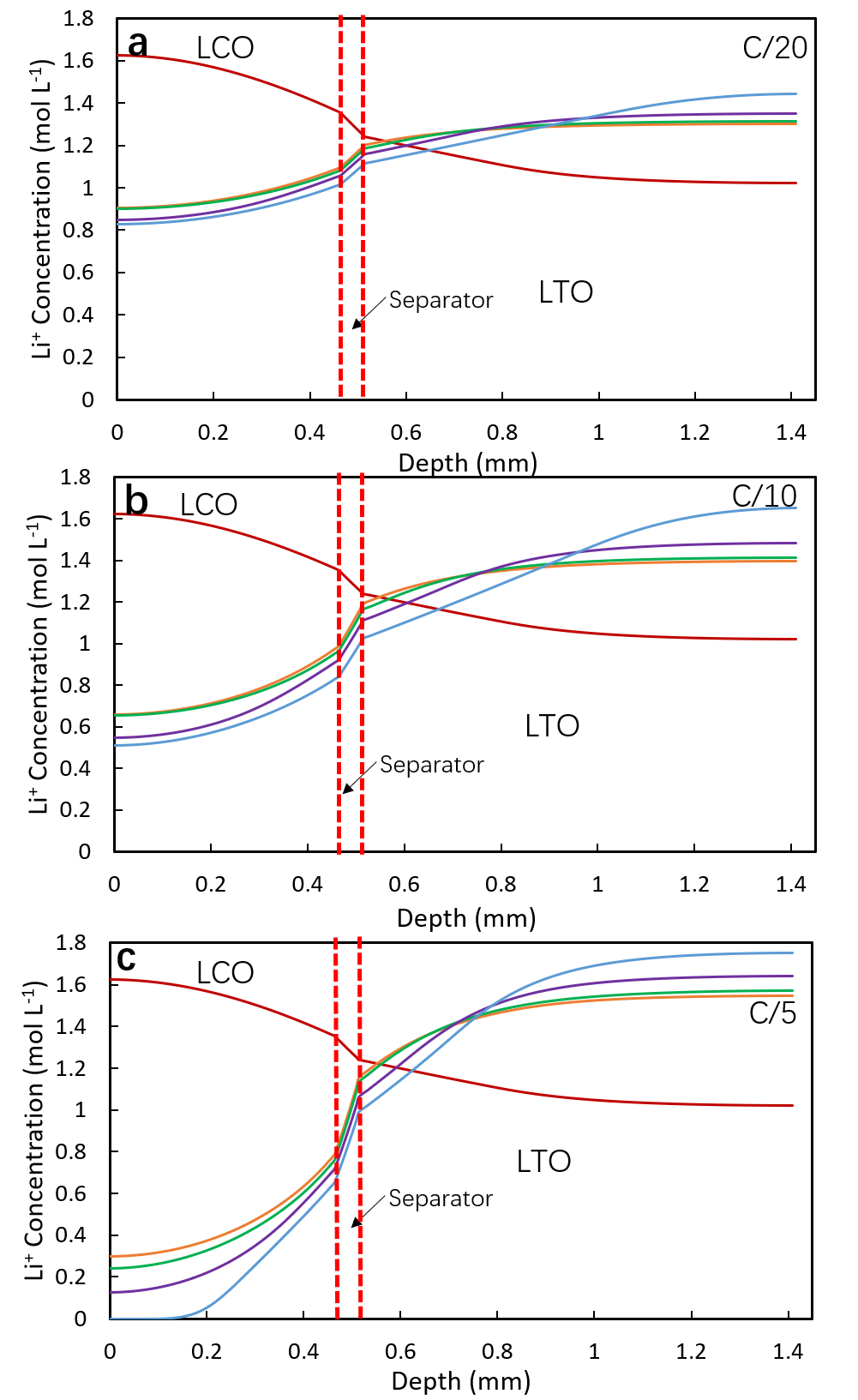


**Fig. S8** Concentration profiles in electrolyte phase for LTO_POR_/LCO cell at (a) C/20, (b) C/10 and (c) C/5 discharge process. The different curves correspond to the different extents of discharge capacity delivered, with the concentrations being shown for 0 % (magenta), 25 % (orange), 50 % (green), 75 % (purple), and 100 % (blue). The profile for 0 % capacity is right at the conclusion of the C/20 charge process.


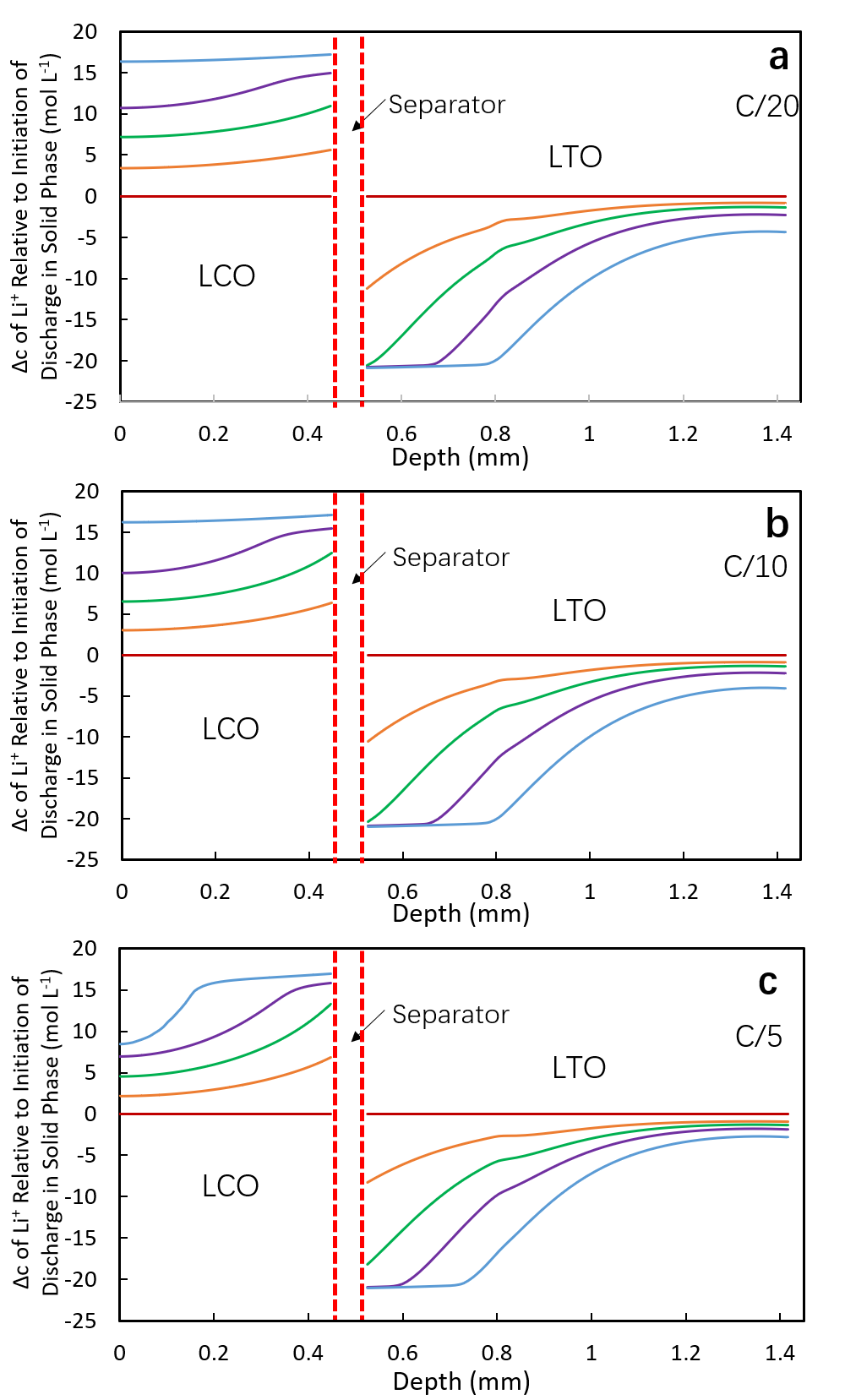


**Fig. S9** Relative concentration profiles in solid phase for LTO_ICE_/LCO cell at (a) C/20, (b) C/10 and (c) C/5. The different curves correspond to the different extents of discharge capacity delivered, with the concentrations being shown for 0 % (magenta), 25 % (orange), 50 % (green), 75 % (purple), and 100 % (blue).


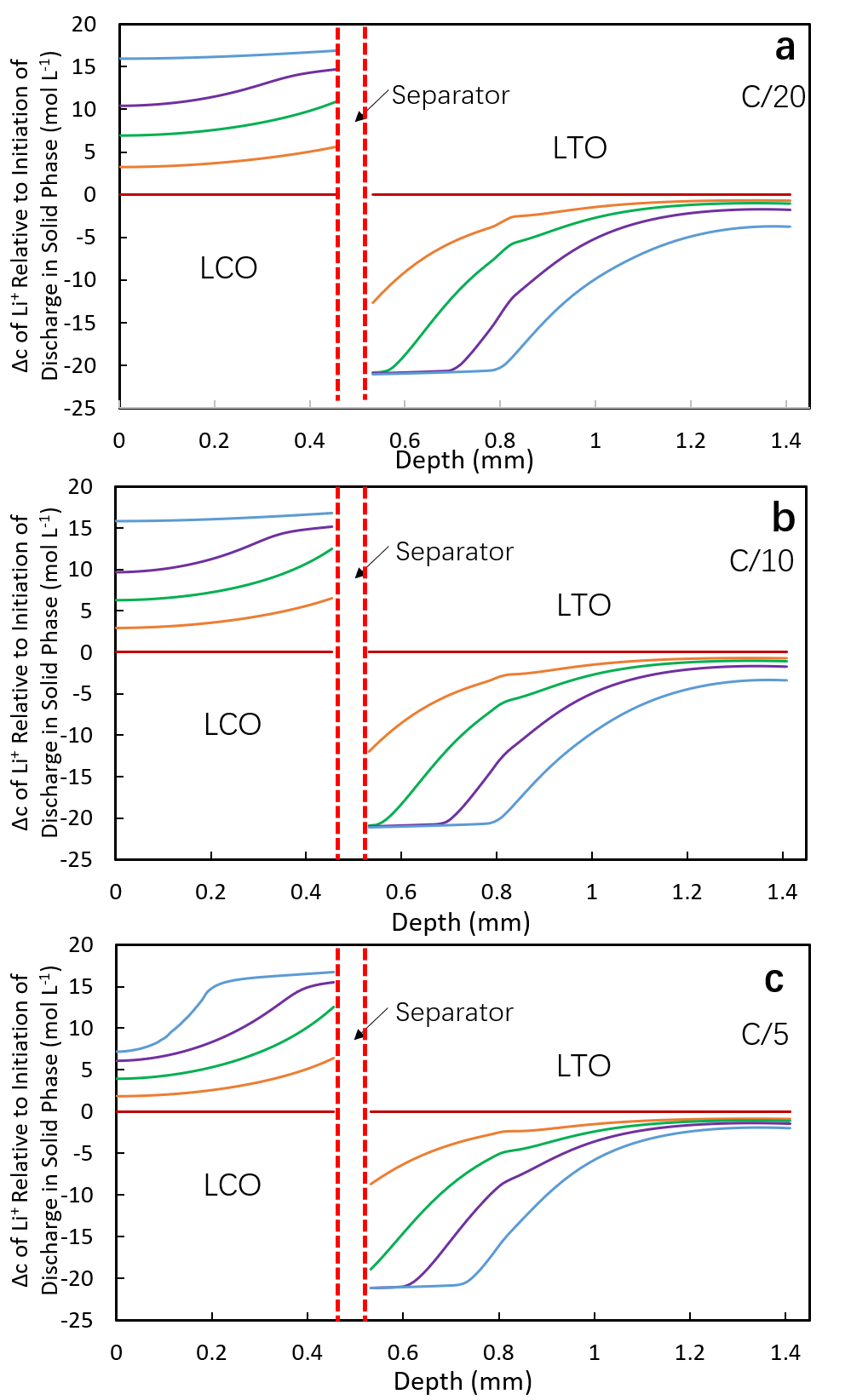


**Fig. S10** Relative concentration profiles in solid phase for LTO_POR_/LCO cell at (a) C/20, (b) C/10 and (c) C/5. The different curves correspond to the different extents of discharge capacity delivered, with the concentrations being shown for 0 % (magenta), 25 % (orange), 50 % (green), 75 % (purple), and 100 % (blue).


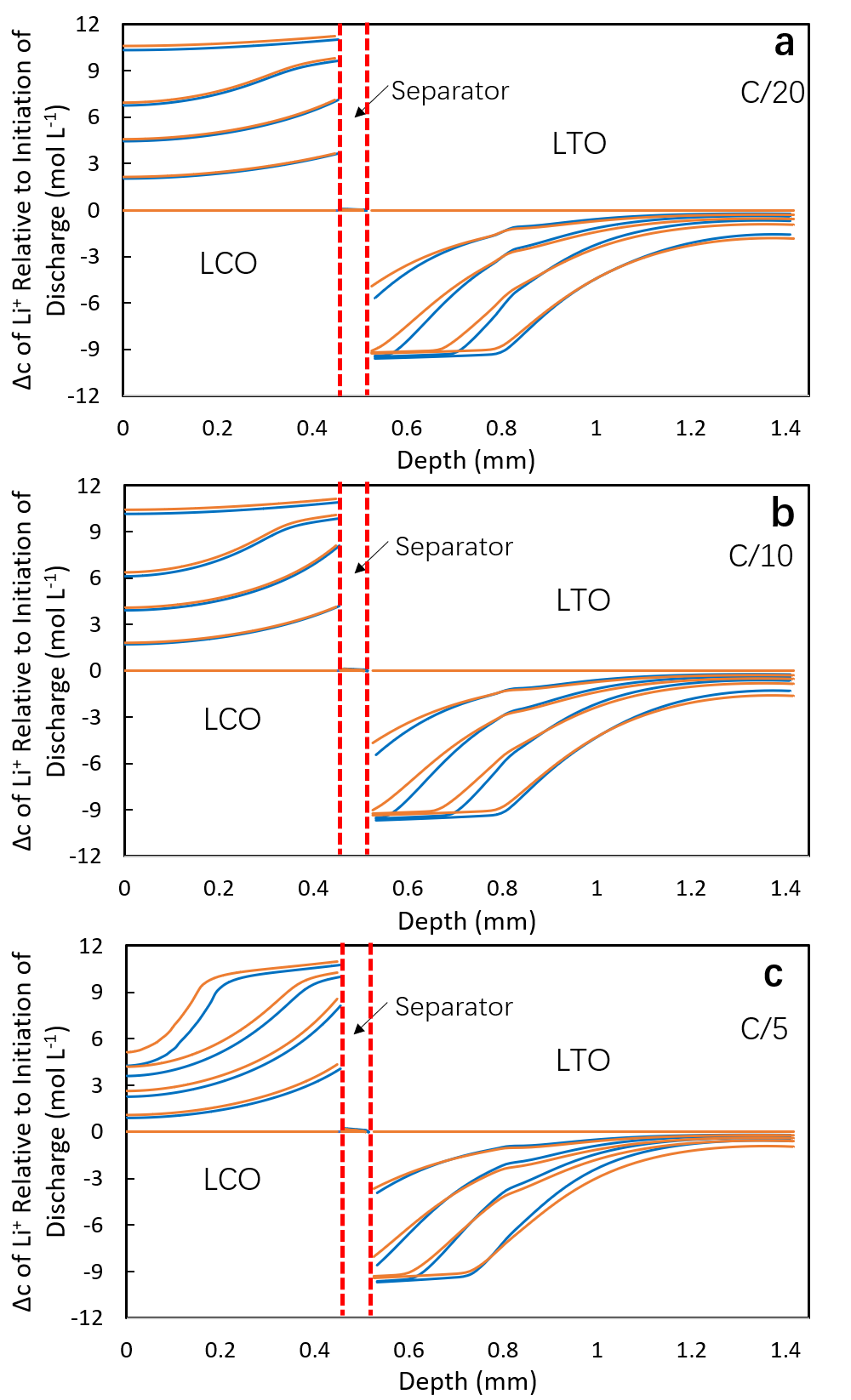


**Fig. S11** Concentration profiles for LTO_ICE_/LCO (orange) and LTO_POR_/LCO (blue) cells that highlights the gradient comparison between two cells. The profiles for each cell are the same as those showed in Fig. 3 and Fig. 4 in main text.


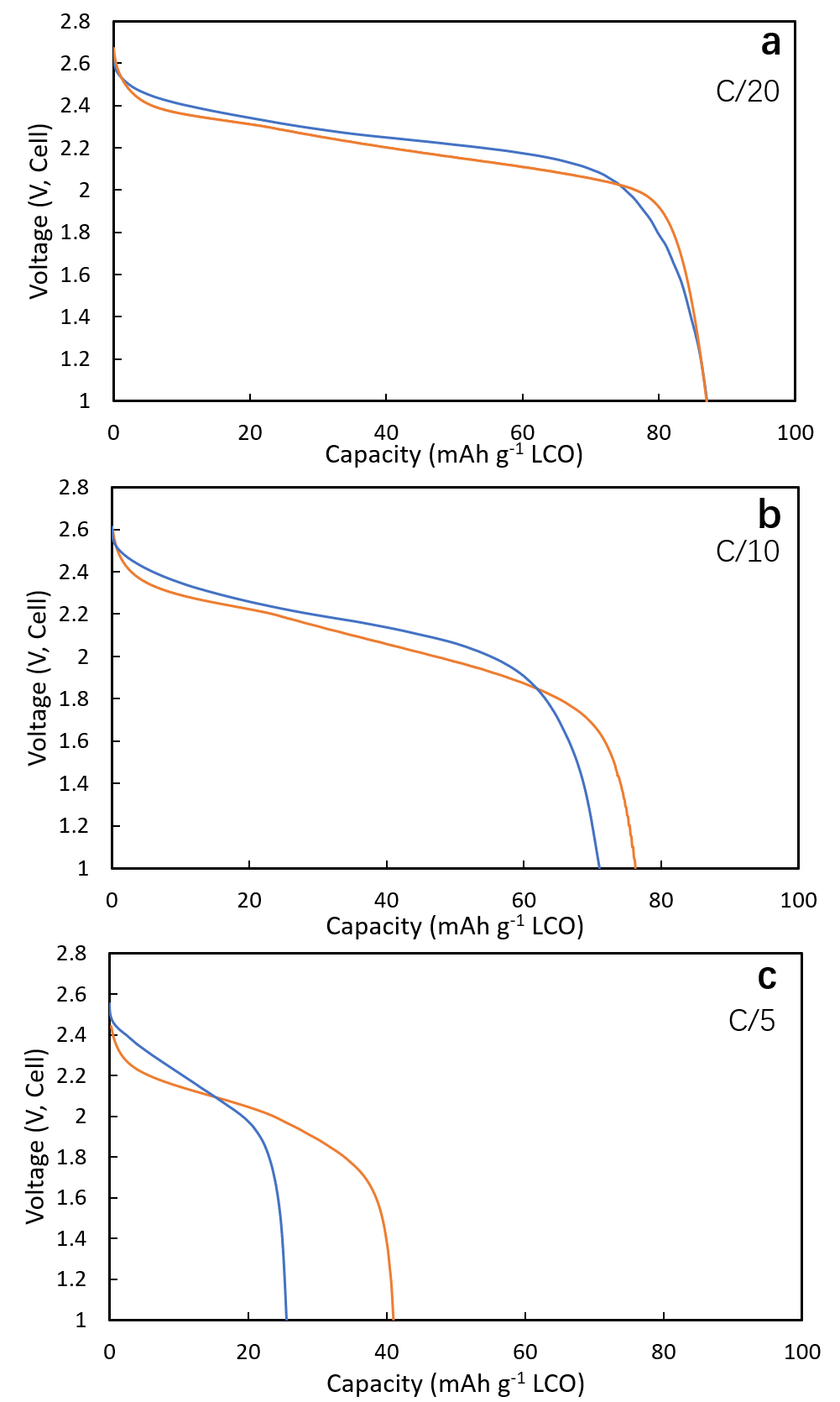


**Fig. S12** Experimental (blue) and calculated (orange) discharge profiles at (a) C/20, (b) C/10 and (c) C/5 for LTO_POR_/LCO cell with α(LTO_POR_)=4.0.


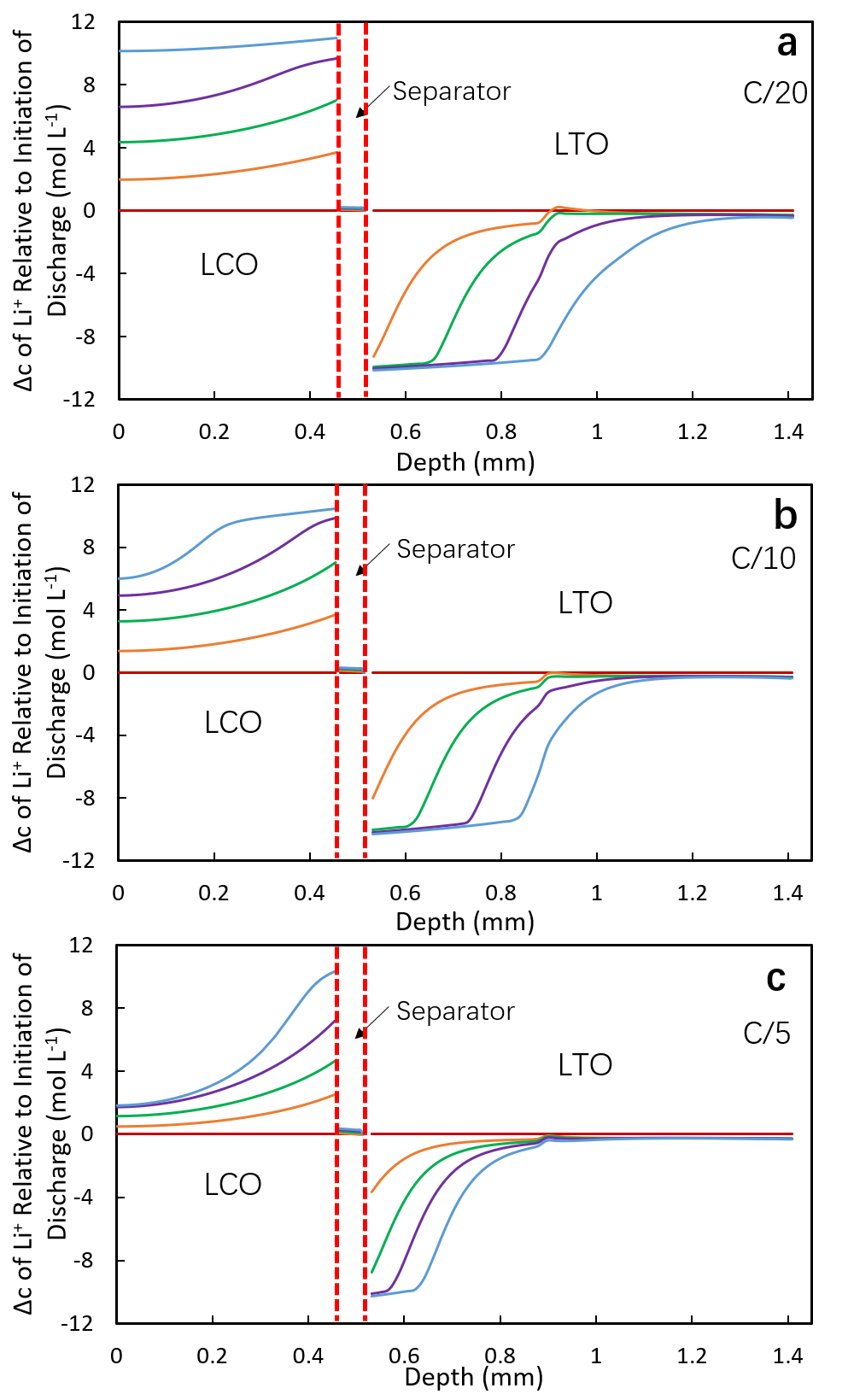


**Fig. S13** Calculated change in total Li^+^ concentration profiles for LTO_POR_/LCO cell when α(LTO_POR_)=4.0 at (a) C/20, (b) C/10 and (c) C/5 discharge process. The different curves correspond to the different extents of discharge capacity delivered, with the concentrations being shown for 0 % (magenta), 25 % (orange), 50 % (green), 75 % (purple), and 100 % (blue).


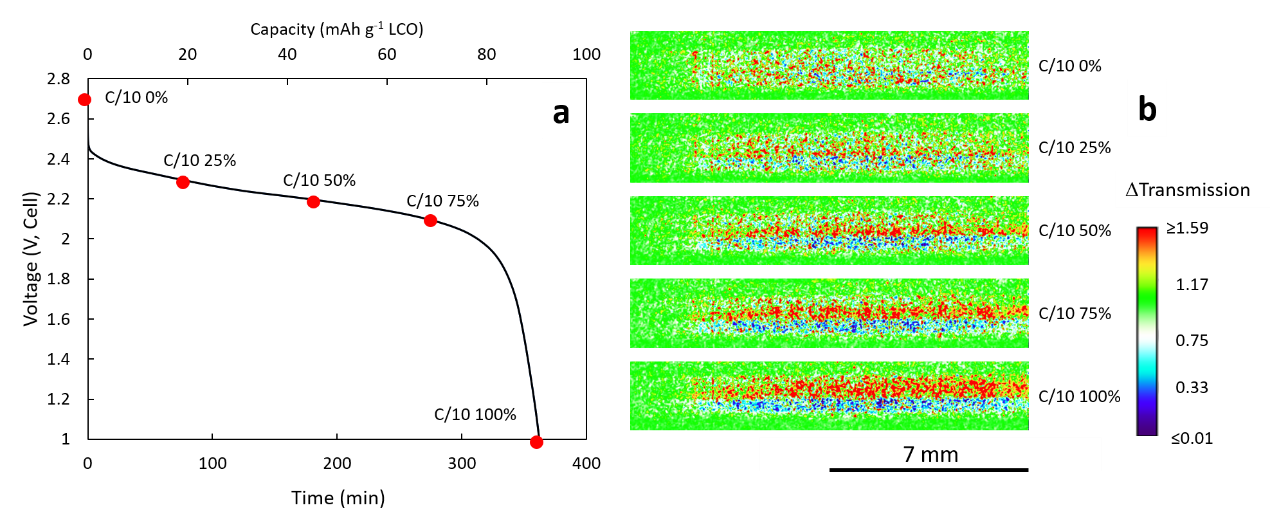


**Fig. S14.** (a) Discharge profiles of LTO_ICE2_/LCO at C/10. (b) Changes in neutron transmission in the electrode region of the cell corresponding to the time points noted in (a).


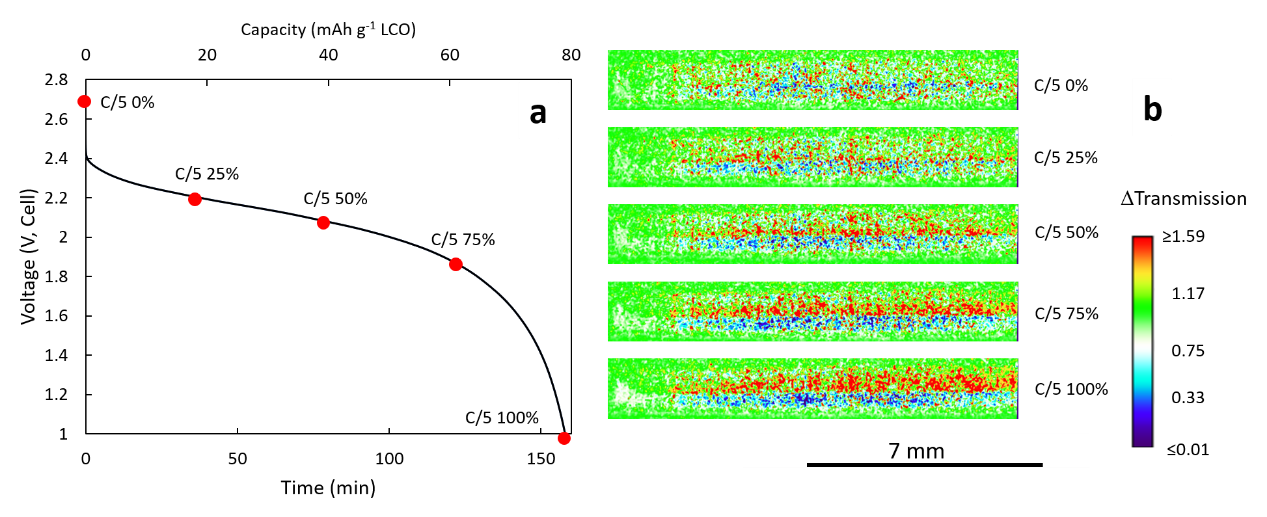


**Fig. S15.** (a) Discharge profiles of LTO_ICE2_/LCO at C/5. (b) Changes in neutron transmission in the electrode region of the cell corresponding to the time points noted in (a).


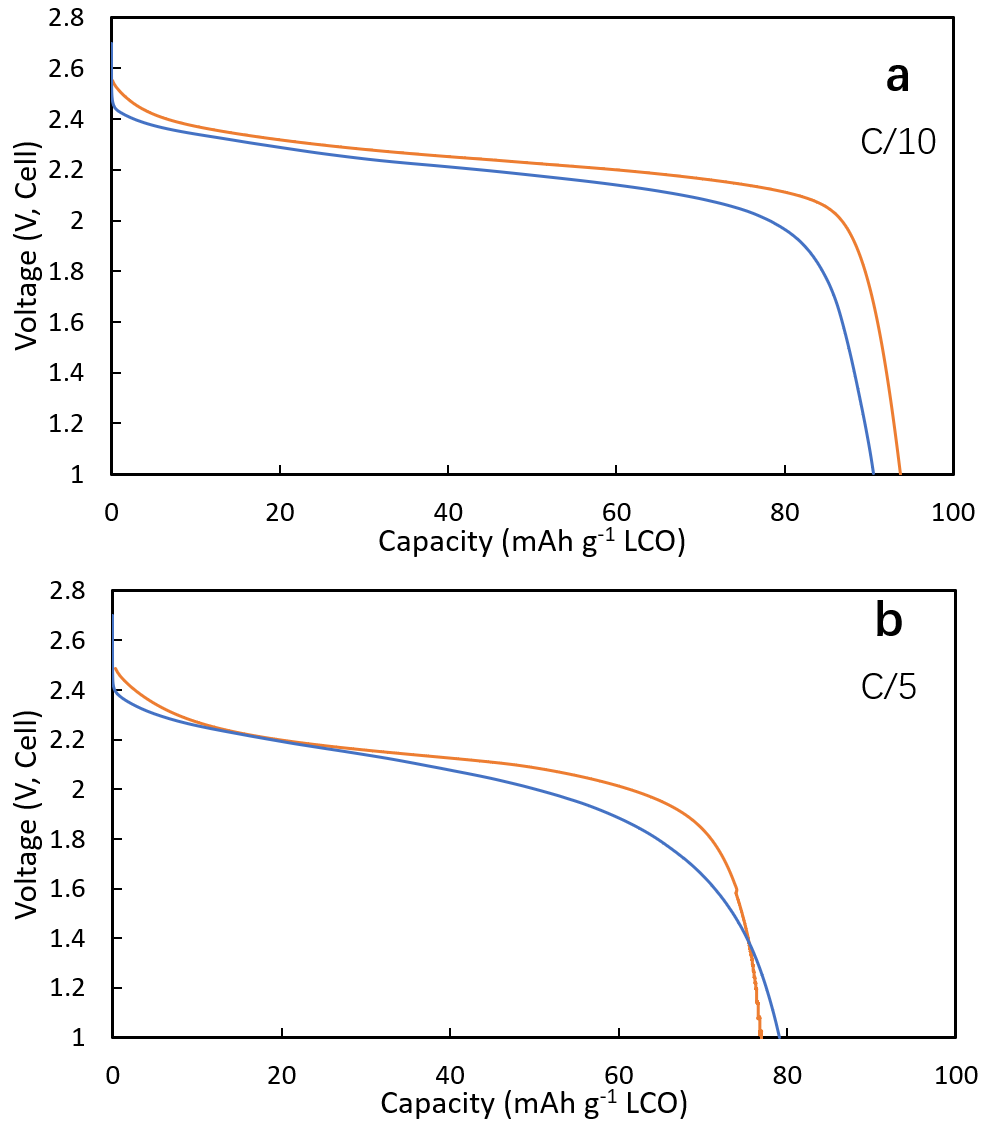


**Fig. S16** Discharge profiles experimentally measured (blue) and calculated (orange) for discharge at (a) C/10 and (b) C/5 for LTO_ICE2_/LCO cell.

**Reference**

1. D. S. Hussey, D. L. Jacobson, M. Arif, K. J. Coakley and D. F. Vecchia, *J Fuel Cell Sci Technol*, **7**, 021024 (2010). [↑](#endnote-ref-1)
2. N. Kardjilov, I. Manke, A. Hilger, M. Strobl, and J. Banhart, *Mater. Today*, **14**, 248-256 (2011).. [↑](#endnote-ref-2)
3. K. Zaghib, M. Simoneau, M. Armand, and M. Gauthier, *J. Power Sources*, **81**, 300–305 (1999). [↑](#endnote-ref-3)
4. J. Xie, N. Imanishi, T. Matsumura, A. Hirano, Y. Takeda, and O. Yamamoto, *Solid State Ion.*, **179**, 362–370 (2008). [↑](#endnote-ref-4)
5. Z. Qi and G. M. Koenig, *J. Power Sources*, **323**, 97–106 (2016). [↑](#endnote-ref-5)
6. Z. Qi and G. M. Koenig, *ChemistrySelect*, **1**, 3992–3999 (2016). [↑](#endnote-ref-6)
7. M. Ménétrier, I. Saadoune, S. Levasseur, and C. Delmas, *J. Mater. Chem.*, **9**, 1135–1140 (1999). [↑](#endnote-ref-7)
8. S. Levasseur, M. Ménétrier, E. Suard, and C. Delmas, *Solid State Ionics*, **128**, 11–24 (2000). [↑](#endnote-ref-8)
9. D. Young, A. Ransil, R. Amin, Z. Li, and Y. M. Chiang, *Adv. Energy Mater.*, **3**, 1125–1129 (2013). [↑](#endnote-ref-9)
10. N. Nitta, F. Wu, J. T. Lee, and G. Yushin, Mater. Today, 18, 252–264 (2015) [↑](#endnote-ref-10)
11. K. Kataoka, Y. Takahashi, N. Kijima, J. Akimoto, and K. I. Ohshima, J. Phys. Chem. Solids, 69, 1454–1456 (2008). [↑](#endnote-ref-11)
12. J. Mao, W. Tiedemann, and J. Newman, ECS Trans., 58, 71–81 (2014). [↑](#endnote-ref-12)
13. B. T. Habte and F. Jiang, Microporous Mesoporous Mater., 268, 69–76 (2018). [↑](#endnote-ref-13)
14. J. Chen, L. Yang, S. Fang, S. I. Hirano, and K. Tachibana, J. Power Sources, 200, 59–66 (2012). [↑](#endnote-ref-14)
15. J. Landesfeind, J. Hattendorff, A. Ehrl, W. A. Wall, and H. A. Gasteiger, *J. Electrochem. Soc.*, 163, A1373-A1387 (2016). [↑](#endnote-ref-15)
16. C. Capiglia, Y. Saito, H. Kageyama, P. Mustarelli, T. Iwamoto, T. Tabuchi, and H. Tukamoto, J. Power Sources, 81, 859–862 (1999). [↑](#endnote-ref-16)
17. A. Nyman, M. Behm, and G. Lindbergh, Electrochim. Acta, 53, 6356–6365 (2008). [↑](#endnote-ref-17)
